# Development and validation of the Home time and Overall survival after Metastatic spine tumor surgery Estimator (HOME score)

**DOI:** 10.1093/noajnl/vdag010

**Published:** 2026-01-23

**Authors:** Husain Shakil, Armaan K Malhotra, Christopher S Lozano, Vishwathsen Karthikeyan, Anne L Versteeg, Jetan H Badhiwala, Arjun Sahgal, Nicolas Dea, Michael G Fehlings, Alexander Kiss, Christopher D Witiw, Donald A Redelmeier, Jefferson R Wilson

**Affiliations:** Division of Neurosurgery, Department of Surgery, University of Toronto, Toronto, Ontario, Canada; Li Ka Shing Knowledge Institute, St Michael’s Hospital, Toronto, Ontario, Canada; Institute of Health Policy Management and Evaluation, University of Toronto, Toronto, Ontario, Canada; Division of Neurosurgery, Department of Surgery, University of Toronto, Toronto, Ontario, Canada; Li Ka Shing Knowledge Institute, St Michael’s Hospital, Toronto, Ontario, Canada; Institute of Health Policy Management and Evaluation, University of Toronto, Toronto, Ontario, Canada; Division of Neurosurgery, Department of Surgery, University of Toronto, Toronto, Ontario, Canada; Li Ka Shing Knowledge Institute, St Michael’s Hospital, Toronto, Ontario, Canada; Institute of Health Policy Management and Evaluation, University of Toronto, Toronto, Ontario, Canada; Division of Neurosurgery, Department of Surgery, University of Toronto, Toronto, Ontario, Canada; Li Ka Shing Knowledge Institute, St Michael’s Hospital, Toronto, Ontario, Canada; Institute of Health Policy Management and Evaluation, University of Toronto, Toronto, Ontario, Canada; Department of Surgery, University of Toronto, Toronto, Ontario, Canada; Division of Neurosurgery, Department of Surgery, University of Toronto, Toronto, Ontario, Canada; Department of Radiation Oncology, Sunnybrook Health Sciences Centre, Toronto, Ontario, Canada; Neurosurgical and Orthopedic Spine Program, Vancouver General Hospital, University of British Columbia, Vancouver, British Columbia, Canada; Division of Neurosurgery, Department of Surgery, University of Toronto, Toronto, Ontario, Canada; Institute of Health Policy Management and Evaluation, University of Toronto, Toronto, Ontario, Canada; Division of Neurosurgery, Department of Surgery, University of Toronto, Toronto, Ontario, Canada; Li Ka Shing Knowledge Institute, St Michael’s Hospital, Toronto, Ontario, Canada; Institute of Health Policy Management and Evaluation, University of Toronto, Toronto, Ontario, Canada; Institute of Health Policy Management and Evaluation, University of Toronto, Toronto, Ontario, Canada; Department of Medicine, Sunnybrook Research Institute, Toronto, Ontario, Canada; Division of Neurosurgery, Department of Surgery, University of Toronto, Toronto, Ontario, Canada; Li Ka Shing Knowledge Institute, St Michael’s Hospital, Toronto, Ontario, Canada; Institute of Health Policy Management and Evaluation, University of Toronto, Toronto, Ontario, Canada

**Keywords:** days at home, home time, prediction model, spinal metastasis, survival

## Abstract

**Background:**

This study reports the development and validation of the Home time and Overall survival after Metastatic spine tumor surgery Estimator (HOME score).

**Methods:**

A population cohort study was conducted, including 2348 adults with spine metastases treated with surgery in the 2005 to 2020 Ontario Cancer registry. HOME score predictions were the likelihood of post-surgery home time of 3-months or less, and overall survival at 6 months, 1 year, and 1.5 years after surgery. Model performance was evaluated using the area under the receiver operating characteristic curve (AUC) for home time predictions, and the concordance index (C-index) for survival. Variable importance was quantified using standardized coefficients.

**Results:**

Mean age was 62.4 years (SD: 12.6) and the most common primary cancer was lung (*N* = 513, 21.9%). Patients treated between 2005 and 2018 were allocated to training, and those treated in 2019-2020 were used as a hold-out test cohort. The final model included 17 items for home time prediction, and 24 items for survival prediction including demographic, comorbid, cancer, and presentation features. Performance of the home time model (AUC: 0.70), and survival model (C-index 0.70, 6-month AUC: 0.73, 1-year AUC: 0.75, 1.5-year AUC: 0.76) was stable across training and testing. Primary cancer origin and history of congestive heart failure (CHF) ranked highest among features impacting outcome predictions.

**Conclusions:**

The HOME score (https://shakilh.shinyapps.io/home_app/) accurately predicts home time and survival for patients with spinal metastases undergoing surgery. Key factors influencing predictions were primary origin, and history of CHF. This represents a significant advancement to patient centered preoperative risk stratification.

Key Points

- HOME score is a reliable prediction model for surgically treated spine metastases
- Home time and survival estimates can be used for patient-centered prognostication
- Primary cancer origin was the most important factor for outcome prediction

Importance of the Study

Patient counseling and selection around surgery for metastatic spinal tumors remain a significant challenge. Relatively few available prognostic tools provide predictions of patient centered outcomes, despite the goal of treatment being patient quality of life. Home time, typically defined as the number of days alive and outside a healthcare institution, is a pragmatic and easily interpretable outcome that is readily available from population health data. This study of 2348 adults treated with surgery, reported the development and validation of a reliable tool for **H**ome time and **O**verall survival after **M**etastatic spine tumor surgery **E**stimation (HOME score). The HOME score (available at: https://shakilh.shinyapps.io/home_app) achieved robust performance on a generalizable training and temporally distinct hold-out testing cohort. Performance was equivalent to the best available survival prognostic tools. Model evaluation found primary cancer origin was the most important factor influencing outcome predictions.

Spinal metastases can cause significant pain and disability for patients with advanced cancer.^1^^,^^2^ Surgery can be an effective treatment for patients,^3^^,^^4^ however, selection of candidates remains a significant challenge^5^^,^^6^ due to difficulty in prognosticating post-operative outcomes for patients suffering advanced cancer.^7–9^

To aid in decision making, a number of prognostic tools have been developed for patients with spinal metastases.^10–20^ Most tools provide estimates of expected survival, despite the primary aim of treatment being quality of life. Home time, typically defined as the number of days alive and outside a healthcare institution, is a pragmatic and easily interpretable outcome that is readily available from population health data. Importantly, it has been validated as a meaningful endpoint for patients with advancer and reliable for those with spinal metastases.^21–24^ Despite this, no existent prognostic tools have integrated this patient centered endpoint into surgical risk assessment.

In this study, we aim to develop and validate an integrated clinical prediction model for home time and survival after surgery for spinal metastases, termed the **H**ome time and **O**verall survival after **M**etastatic spine tumor surgery **E**stimator (HOME score).

## Methods

This study was approved by the Unity Health Toronto research ethics board (REB 21-145) and the Privacy and Legal Office of the ICES, formerly known as the Institute of Clinical Evaluative Sciences, who waived individual patient consent. In addition, this study was conducted and reported in accordance with the TRIPOD+AI guidelines for reporting clinical prediction models using regression or machine learning methods.^25^

### Data

Patient health records were retrieved from ICES,^26^ an independent and nonprofit research institute whose legal status under Ontario’s health information privacy law allows it to collect and analyze health care and demographic data (without expressed consent) for health system evaluation and improvement. Data used for development, validation, and testing were acquired through provincial database linkage at ICES as described in prior studies.^1^^,^^27^ In brief, we used unique ICES identifiers to link records from the Ontario Cancer Registry (OCR), the Ontario Health Insurance Plan (OHIP) database, and the Cancer Care of Ontario Activity Level Reporting database to identify patients undergoing surgery for a spine metastasis between years 2005 through 2020. Additional clinical records for model predictors and outcomes were retrieved from the Registered Person’s Database (RPDB), the Canadian Institute for Health Information Discharge Abstract Database (DAD), Same-day Surgeries Database, National Ambulatory Care Reporting System (NACRS), the Continuing Care Reporting System (CCRS), National Rehabilitation Reporting System, Ontario Mental Health Reporting System, and Home Care Database.

### Participants and Data Preparation

We included all patients aged over 18 years with spinal metastases treated with surgery.^28–30^ Surgical patients were identified using OHIP fee codes corresponding to a spinal surgery within 1 year of their diagnosis of spinal metastasis linked to a neoplastic diagnosis code (Supplementary Table 1—see online supplementary material). Patients with loss of OHIP eligibility on or prior to their date of surgery were excluded. All patients either surviving the two-year follow-up period, or with a recorded death date within the 2-year follow-up period were included. Patients lost to follow-up due to loss of OHIP ineligibility were excluded. Patients treated between 2005 and 2018 were allocated to the training cohort, and the remaining treated from 2019 to 2020 were allocated to the hold-out testing cohort (Supplementary Figure 1—see online supplementary material for a color version of this figure). To ensure no data leak, all records from the training and testing cohort were separated within distinct files and handled in separate sessions.

### Outcomes

Home time and survival were co-primary outcomes. Home time was defined as the total number of days spent alive outside of a healthcare institution within a 2-year postoperative follow-up period, as described in prior studies.^21^^,^^24^^,^^31^ Institutional days were identified by accounting for inpatient hospitalizations, same-day surgeries, emergency department visits, admissions within the CCRS, long-term care stays, inpatient rehabilitation, inpatient mental health, and home care records.^32–34^ Both institutional days, and days after the date of death did not contribute to home time. We classified patients as having unfavorable home time if they spent less than or equal to 3 months at home. This was not restricted to 3 contiguous months, and any patient with more than 90 days total at home after a 2-year post-surgery window were not considered to have unfavorable home time. Three months was chosen as the dichotomy as this has historically been used as the minimum expected survival time required for patients to be offered surgery.^35–37^ Survival after surgery was determined using all-cause mortality with vital statistics from the RPDB. Model predictions were provided as overall survival probability at 6 months, 1 year, and 1.5 years.

### HOME Score Features

Candidate features selected for inclusion were determined based on literature review,^10^^,^^11^^,^^38^^,^^39^ and availability within clinical records. Features were broadly categorized as demographic, comorbid, cancer related, and presentation related. Demographic features included age, sex, home location, distance from and home to nearest cancer center. We included comorbidities relevant to operative risk stratification^40^ such as preexisting history of congestive heart failure (CHF), stroke, cardiovascular disease, pulmonary disease, liver failure, peptic ulcer disease (PUD), diabetes, hematologic disorders, endocrine disorders, and psychiatric disorders. Patient comorbidities were identified using validated codes.^41^^,^^42^ Cancer-related features were determined using the OCR, which has been validated as a complete cancer registry for Ontario.^43^ The primary origin of cancer, years since primary diagnosis, intent of most recent chemotherapy, number of lines of chemotherapy trialed, intent of most recent nonspinal radiation, courses of nonspinal radiation trialed, the number of body metastases, the presence of brain or visceral metastases, the affected spinal level, and receipt of prior spinal radiation were included as cancer-related features. Presenting features included the presence of paralysis or spinal cord injury, admission to an intensive care unit (ICU) within 1 year prior, and the number of emergency department visits within 3-months prior to surgery were included as presenting features (Supplementary Methods, Supplementary Table 1—see online supplementary material).

### Sample Size

Our sample size was determined based on a minimum of 20 events per variable for training a standard logistic or survival regression model. After data preparation, candidate features required 43 coefficients for modeling (Supplementary Table 1—see online supplementary material). During a 2-year follow-up, the expected overall survival for the patient cohort was 20%.^30^^,^^36^ Accordingly, the minimum sample size for our training cohort was pre-specified to be 1075 patients.

### Analytical Methods

Statistical analyses were performed using R Studio (R Foundation for Statistical Computing, Vienna, Austria) version 4.2.1 with a significance level of *P* = .05 for two-tailed tests. Descriptive cohort statistics were reported as mean, and standard deviation (SD) for continuous variables and count with percentages for categorical variables. Descriptive statistics for home time was provided with a 1000 iteration bootstrap of the median, and a Kaplan–Meier estimator for median survival. Univariable comparisons between the training and hold-out testing cohort were done using Welch’s t-test for continuous variables and the Chi-squared test for binary and categorical variables.

Missingness was assessed as less than 20% for all variables and outcomes (Supplementary Tables 2 and 3—see online supplementary material). For the training cohort missing records were imputed with predictive mean matching though multiple imputation over 10 iterations, as described in prior studies.^44^ We included only complete cases within our hold-out test cohort, to ensure testing was conducted exclusively on real clinical records.

We trained 5 model architectures for the dichotomous home time outcome, and 5 architectures for the survival outcome (Supplementary Methods—see online supplementary material). In brief, a linear logistic, nonlinear logistic, linear least absolute shrinkage operator (LASSO) logistic, nonlinear LASSO logistic, and extreme gradient boosted binary classifiers were trained to predict the dichotomous home time outcome. A linear cox proportional hazard (CPH), nonlinear CPH, linear LASSO CPH, nonlinear LASSO CPH, and gradient boosted survival models were trained to predict post-operative survival. The regularization parameter within LASSO models was used to eliminate variables with shrunken coefficients.

Within the training cohort, home time classifiers were evaluated using the area under the receiver operating characteristic (AUC) on the test fold of a 10-fold cross-validation. Similarly, survival models were evaluated using the concordance-index. We used the 95% confidence interval of performance metrics to select the architecture with the highest performance and least number of variables for computational parsimony.

**Figure 1.**
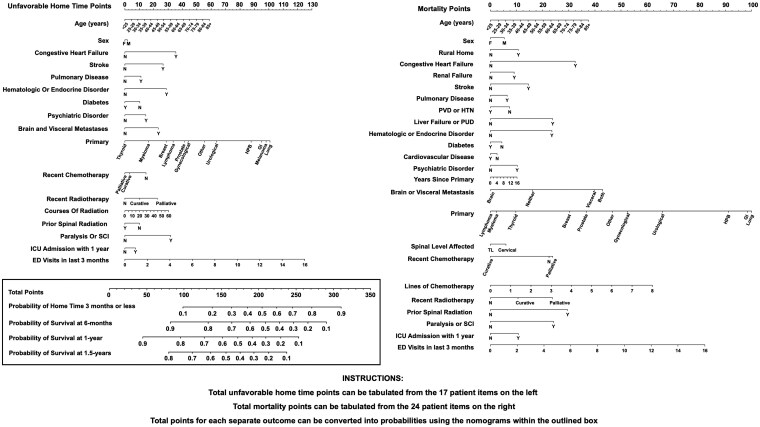
Integrated prediction model of home time 3 months or less (unfavorable home time) and overall survival after surgery for patients with spine metastases. Total unfavorable home time points can be tabulated from the 17 patient items on the left, and total mortality points can be tabulated from the 24 patient items on the right. The nomograms within the black traced rectangle can be used to convert the total unfavorable home time points into a predicted probability of 3-months or less total home time after surgery for a metastatic spine tumor. As well, total mortality points can be converted to a predicted probability of survival at 6 month, 1 year, and 1.5 year. A greater number of points on each nomogram portends a higher likelihood of unfavorable home time and shorter survival, respectively. Abbreviations: ED, emergency department; F, female; GI, gastrointestinal; HPB, hepatobiliary; HTN, hypertension; ICU, intensive care unit; M, male; N, no; PUD, peptic ulcer disease; PVD, peripheral vascular disease; SCI, spinal cord injury; TL, thoracolumbar; Y, yes.

The final architectures selected were re-trained on the entire training cohort (without cross-validation) to develop the HOME score, which was externally tested on the hold-out cohort. On the test cohort we assessed the AUC, Brier score and calibration of home time classification; and C-index, time dependent AUC, and time-dependent calibration of the survival prediction at 6 months, 1 year, and 1.5 years. Errors were evaluated by comparing records between cases correctly and incorrectly classified with respect to home time or survival (Supplementary Methods—see online supplementary material). Variable importance was assessed using standardized coefficients as previously described.^44^ We implemented graphical nomograms of the HOME score for clinical application using the *rms* version 7.0-0 package. Figure 1 illustrates our graphical depiction of the HOME score which functions as 2 complementary nomograms for predicting unfavorable home time (3 months or less after surgery) and overall survival at 6 months, 1 year, and 1.5 years. Each nomogram translates a multivariable regression model (logistic or CPH) into a visual tool that assigns points to individual patient characteristics. Figure 1 assigns points for each predictor variable using a corresponding “Unfavourable Home Time Points” scale and a similar “Mortality Points” scale. Individual points are then summed to generate a total points score for each outcome, ranging from 0 to 350. Each respective set of points (unfavorable home time points or mortality points) can be mapped directly onto a probability scale using the nomograms in the boxed portion of Figure 1. Thus, once patient variables are tallied, clinicians can quickly read off the nomogram to obtain individualized estimates of both home time and survival likelihood, facilitating rapid clinical decision-making. The theoretical minimum number of home time points is zero, corresponding to a less than 10% predicted probability of 3 months or less home time after surgery, while the theoretical maximum is 350 corresponding to a greater than 90% predicted probability of 3 months or less home time after surgery. Similarly, the theoretical minimum number of mortality points is zero, corresponding to a greater than 90% predicted probability of survival at 6 months and 1 year, and greater than 80% survival at 1.5 years. The theoretical maximum number of mortality points is 350 corresponding to a less than 10% predicted probability of survival at 6 months, 1 year, and 1.5 years.

As a secondary alternative, we created a free online version of the HOME score (https://shakilh.shinyapps.io/home_app/) that can also be used to generate postsurgery survival and home time predictions. The web application was generated using *shiny* version 1.10.

Using the HOME score nomogram, home time and survival for patients scoring 100-150 total unfavorable home time points and mortality points, respectively, were compared to individuals scoring 200-250. As well we compared HOME score unfavorable home time points and mortality points among patients within the lowest and highest quartiles of postoperative home time and survival, respectively (Supplementary Methods—see online supplementary material).

## Results

### Overview

We identified 2347 eligible patients with spinal metastases treated with surgery. Mean age was 62.4 years (SD 12.6) and 975 (41.5%) of patients were female. The 5 most common primary cancers were lung (*N* = 513, 21.9%), breast (*N* = 295, 12.6%), myeloma (292, 12.4%), prostate (*N* = 256, 10.9%), and urological (*N* = 235, 10.0%). Most patients were treated with decompression and fusion (*N* = 1472, 62.7%) through a posterior approach (*N* = 1713, 73.0%). There were 695 (29.6%) patients treated for cervical spine metastases, 1598 (68.1%) patients treated for thoracolumbar metastases, and the remaining 54 (2.3%) were not otherwise specified. There were 648 (27.6%) patients treated with neoadjuvant radiation prior to surgery, and 1239 (52.8%) patients treated with postsurgical adjuvant radiation. Median survival was 326 days (95% CI: 301-363 days), and median home time was 301 days (95% CI: 260–332 days) over a 2-year postoperative horizon.

There were 2035 (86.7%) patients allocated to the training cohort, and 265 (11.3%) patients allocated to hold-out testing. We found significant differences between cohorts with respect to rates of comorbidities, years since diagnosis, types of primary cancer diagnosis, and cancer treatments received prior to surgery (Table 1). Median survival for the training cohort was 326 days (95% CI: 300-368 days), and 317 days (95% CI: 250-450 days) for the testing cohort. Median home time was 286 days (95% CI: 246-312 days) for the training cohort, and 356 days (95% CI: 110-478 days) for patients allocated to testing. Six hundred twenty-five (30.7%) patients in the training cohort and 80 (30.2%) patients in testing had unfavorable home time.

**Table 1.** Comparison of characteristics of the training and testing cohort.

	**Training** *N* = 2035^a^	**Hold-out Testing** *N* = 265^a^	***P*-value** ^b^
Demographics			
Age (years)			.010
20-59	789 (38.8%)	78 (29.4%)	
60-79	1,125 (55.3%)	166 (62.6%)	
80 or greater	121 (5.9%)	21 (7.9%)	
Sex			.2
**Female**	834 (41.0%)	121 (45.7%)	
**Male**	1,201 (59.0%)	144 (54.3%)	
Rural Home	248 (12.2%)	33 (12.5%)	>.9
Distance from home to nearest cancer centre (Km)	30.1 (46.3)	28.0 (51.7)	.5
Neighborhood Socioeconomic Quintile			.21
1	440 (21.8%)	41 (15.5%)	
2	392 (19.4%)	40 (15.2%)	
3	406 (20.1%)	58 (22.0%)	
4	382 (18.9%)	61 (23.1%)	
5	396 (19.6%)	64 (24.2%)	
Prior comorbidities			
Stroke	49 (2.4%)	15 (5.7%)	.005
Congestive heart failure	34 (1.7%)	7 (2.6%)	.4
Cardiovascular disease	168 (8.3%)	39 (14.7%)	<.001
Pulmonary disease	149 (7.3%)	21 (7.9%)	.8
Liver failure or peptic ulcer disease	45 (2.2%)	11 (4.2%)	.086
Renal failure	38 (1.9%)	6 (2.3%)	.8
Peripheral vascular disease or hypertension	430 (21.1%)	64 (24.2%)	.3
Diabetes	225 (11.1%)	42 (15.8%)	.029
Hematologic or endocrine disorder	244 (12.0%)	56 (21.1%)	<.001
Psychiatric disorders	84 (4.1%)	8 (3.0%)	.5
Cancer features			
Primary			.049
Lung	455 (22.4%)	49 (18.5%)	
Myeloma	262 (12.9%)	29 (10.9%)	
Breast	241 (11.8%)	45 (17.0%)	
Prostate	206 (10.1%)	41 (15.5%)	
Other	216 (10.6%)	26 (9.8%)	
Urological	206 (10.1%)	22 (8.3%)	
Gastrointestinal	141 (6.9%)	22 (8.3%)	
Lymphoma	105 (5.2%)	8 (3.0%)	
Hepatobiliary	61 (3.0%)	<6 (<2%)	
Melanoma	61 (3.0%)	<6 (<2%)	
Gynecological	50 (2.5%)	8 (3.0%)	
Thyroid	31 (1.5%)	<6 (<2%)	
Intent of most recent chemotherapy			<.001
No prior chemotherapy	1,407 (69.1%)	137 (51.7%)	
Curative	200 (9.8%)	46 (17.4%)	
Palliative	428 (21.0%)	82 (30.9%)	
Lines of chemotherapy received			.5
0	1,714 (84.2%)	214 (80.8%)	
1	271 (13.3%)	40 (15.1%)	
2 or greater	50 (24.6%)	11 (4.2%)	
Intent of most recent nonspinal radiation			<.001
No prior radiotherapy	1,461 (71.8%)	158 (59.6%)	
Curative	296 (14.5%)	62 (23.4%)	
Palliative	278 (13.7%)	45 (17.0%)	
Courses of nonspinal radiation received	1.1 (5.4)	1.7 (6.9)	.2
Neoadjuvant spinal radiation	568 (27.9%)	56 (21.1%)	.024
Adjuvant spinal radiation	1,055 (51.8%)	170 (64.2%)	<.001
Number of metastases			<.001
None	1,668 (82.0%)	259 (97.7%)	
Single	106 (5.2%)	<6 (<2%)	
Multiple	261 (12.8%)	<6 (<2%)	
Brain or visceral metastases			.027
None	1,893 (93.0%)	259 (97.7%)	
Visceral alone	110 (5.4%)	<6 (<2%)	
Brain alone	20 (1.0%)	0 (0.0%)	
Brain and visceral	12 (0.6%)	<6 (<2%)	
Spinal metastases level			.8
Cervical	608 (29.9%)	77 (29.1%)	
Thoracolumbar	1,427 (70.1%)	188 (70.9%)	
Years since primary diagnosis	1.6 (2.6)	3.1 (4.1)	<.001
Presenting features			
Paralysis or spinal cord injury	501 (24.6%)	71 (26.8%)	.5
ICU admission in prior year	306 (15.0%)	47 (17.7%)	.3
Number of ED visits in 3-months prior	1.9 (1.7)	2.1 (1.8)	.043
Treatment and outcomes			
Year of surgery			<.001
**2005-2008**	421 (20.7%)	0 (0.0%)	
**2009-2012**	538 (26.4%)	0 (0.0%)	
**2013-2016**	679 (33.4%)	0 (0.0%)	
**2017-2018**	397 (19.5%)	0 (0.0%)	
**2019-2020**	0 (0.0%)	265 (100.0%)	
Type of surgery			.4
Decompression alone	523 (28.0%)	78 (32.0%)	
Decompression with fusion	1,285 (68.8%)	159 (65.2%)	
Instrumented fusion alone	61 (3.3%)	7 (2.9%)	
Surgical approach			<.001
Anterior	162 (8.7%)	7 (2.9%)	
Combined	234 (12.6%)	17 (7.1%)	
Posterior	1,461 (78.7%)	216 (90.0%)	
Postoperative follow-up (days)	818.4 (1,080.5)	258.3 (211.4)	<.001
Died	1,650 (81.1%)	142 (53.6%)	<.001
Home time (days)	357.3 (290.4)	385.5 (305.7)	.2
Home time 3 months or less	625 (30.7%)	80 (30.2%)	>.9

Abbreviations: ED, emergency department; ICU, intensive care unit.

a n (%) for categorical variables; Mean (SD) for continuous variables.

b Pearson’s Chi-squared test; Welch two sample *t*-test.

### HOME Score Development and Validation

Cross-validation found no differences in the performance between the linear, nonlinear, linear LASSO, and nonlinear LASSO architectures (Table 2). The linear LASSO architecture had 12 terms eliminated for the home time model through regularization (Supplementary Table 4—see online supplementary material) and was selected for the HOME score (cross-validated AUC on test fold 0.72, 95% CI: 0.70-0.73). The linear LASSO architecture had 4 terms eliminated for the survival model (Supplementary Table 4—see online supplementary material) and was selected (cross-validated AUC on test fold 0.69, 95% CI: 0.67-0.70). After retraining on the entire training cohort, hold-out testing found an AUC of 0.70 and Brier score 0.19 for prediction of home time of 3-months or less, and a C-index of 0.70 for survival prediction. Moreover, on hold-out testing, time-dependent AUC of overall survival prediction at 6 months, 1 year, and 1.5 years were 0.73, 0.75, and 0.76, respectively. Analogous time dependent Brier scores were 0.19, 0.20, and 0.20, respectively. Inspection of calibration plots found good calibration for home time and survival prediction across deciles of overall survival (Supplementary Figure 2—see online supplementary material for a color version of this figure). An integrated nomogram of the trained and validated HOME score is depicted in Figure 1, for tabulating risk of unfavorable home time and survival probability from preoperative patient covariates.

**Table 2.** Performance of various architectures during training, cross-validation, and hold-out testing.

	Training	Cross-validation (95% CI)	Hold-Out Test
**Home Time Models—AUC**			
Linear	0.74	0.71 (0.69-0.73)	0.70
Nonlinear	0.76	0.71 (0.69-0.74)	0.72
LASSO linear	**0.74**	**0.72 (0.70-0.73)**	**0.70**
LASSO Nonlinear	0.75	0.72 (0.70-0.74)	0.72
XGBoost	0.69	0.68 (0.65-0.70)	0.65
**Survival Models—C-Index**			
Linear	0.70	0.69 (0.67-0.70)	0.69
Nonlinear	0.71	0.69 (0.68-0.70)	0.69
LASSO linear	**0.70**	**0.69 (0.67-0.70)**	**0.70**
LASSO nonlinear	0.70	0.69 (0.69-0.70)	0.69
Gradient boosted	0.74	0.70 (0.68-0.71)	0.70

The bolded architectures were selected for the final model.

Abbreviations: AUC, area under the receiver operative characteristic curve; C-index, Concordance index; LASSO, least absolute shrinkage and selection operator; XGBoost, Extreme gradient boosting.

### Model Evaluation

On hold-out testing, 101 (38.1%) cases were misclassified at the optimal threshold probability (Supplementary Figure 3—see online supplementary material for a color version of this figure). Among the 101 cases, 43 (42.6%) were misclassified for both home time and 6-month survival, 30 (29.7%) for home time alone, and 28 (27.7%) for 6-month survival alone. Comparison of misclassified patients to the remaining hold-out test cohort, found misclassified patients differed significantly with respect to systemic chemotherapy and radiation therapy received prior to undergoing surgery (Table 3). We also found significantly higher rates of lung cancer (25.7% vs 14.0%, *P* = .006 chi-squared) within the misclassified subgroup.

**Table 3.** Comparisons between misclassified and correctly classified cases in the hold-out cohort of patients.

	**Correctly Classified** *N* = 164^a^	**Misclassified** *N* = 101^a^	***P*-value** ^b^
Age (years)			.7
20-59	51 (31.1%)	27 (26.7%)	
60-79	101 (61.6%)	65 (64.4%)	
80 or greater	12 (7.3%)	9 (8.9%)	
Sex			.7
**Female**	77 (47.0%)	44 (43.6%)	
**Male**	87 (53.0%)	57 (56.4%)	
Rural home	21 (12.8%)	12 (11.9%)	>.9
Distance from home to nearest cancer center (Km)	30.4 (62.0)	24.2 (27.7)	.3
Neighborhood socioeconomic quintile			.3
1	41 (25.0%)	23 (23.0%)	
2	39 (23.8%)	22 (22.0%)	
3	31 (18.9%)	27 (27.0%)	
4	23 (14.0%)	17 (17.0%)	
5	30 (18.3%)	11 (11.0%)	
Stroke	6 (3.7%)	9 (8.9%)	.13
Congestive heart failure	<6 (<4%)	<6 (<6%)	.9
Cardiovascular disease	24 (14.6%)	15 (14.9%)	>.9
Pulmonary disease	13 (7.9%)	8 (7.9%)	>.9
Liver failure or peptic ulcer disease	7 (4.3%)	<6 (<6%)	>.9
Renal failure	<6 (<4%)	<6 (<6%)	>.9
Peripheral vascular disease or hypertension	43 (26.2%)	21 (20.8%)	.4
Diabetes	24 (14.6%)	18 (17.8%)	.6
Hematologic or endocrine disorder	32 (19.5%)	24 (23.8%)	.5
Psychiatric disorders	<6 (<4%)	<6 (<6%)	>.9
Primary			.006
Lung	23 (14.0%)	26 (25.7%)	
Breast	34 (20.7%)	11 (10.9%)	
Prostate	30 (18.3%)	11 (10.9%)	
Myeloma	23 (14.0%)	6 (5.9%)	
Other	15 (9.1%)	11 (10.9%)	
Gastrointestinal	13 (7.9%)	9 (8.9%)	
Urological	11 (6.7%)	11 (10.9%)	
Gynecological	<6 (<4%)	6 (5.9%)	
Lymphoma	6 (3.7%)	<6 (<6%)	
Hepatobiliary	<6 (<4%)	<6 (<6%)	
Melanoma	<6 (<4%)	<6 (<6%)	
Thyroid	<6 (<4%)	<6 (<6%)	
Intent of most recent chemotherapy			.9
No prior chemotherapy	83 (50.6%)	54 (53.5%)	
Curative	30 (18.3%)	16 (15.8%)	
Palliative	51 (31.1%)	31 (30.7%)	
Lines of chemotherapy received			.4
0	132 (80.5%)	82 (81.2%)	
1 or greater	32 (19.5%)	19 (18.8%)	
Intent of most recent nonspinal radiation			.070
No Prior radiotherapy	96 (58.5%)	62 (61.4%)	
Curative	45 (27.4%)	17 (16.8%)	
Palliative	23 (14.0%)	22 (21.8%)	
Courses of nonspinal radiation received	1.6 (6.8)	1.8 (7.2)	.9
Number of metastases			.5
None	160 (97.6%)	99 (98.0%)	
Single	<6 (<4%)	<6 (<6%)	
Multiple	<6 (<4%)	<6 (<6%)	
Brain or visceral metastases			.7
None	160 (97.6%)	99 (98.0%)	
Visceral alone	<6 (<4%)	<6 (<6%)	
Brain alone	<6 (<4%)	<6 (<6%)	
Brain and visceral	<6 (<4%)	<6 (<6%)	
Spinal metastases level			.4
Cervical	51 (31.1%)	26 (25.7%)	
Thoracolumbar	113 (68.9%)	75 (74.3%)	
Years since primary diagnosis	3.3 (4.1)	2.7 (4.0)	.3
Neoadjuvant spinal radiation	30 (18.3%)	26 (25.7%)	.2
Paralysis or spinal cord injury	44 (26.8%)	27 (26.7%)	>.9
ICU admission in prior year	29 (17.7%)	18 (17.8%)	>.9
Number of ED visits in 3-months prior	2.0 (1.7)	2.4 (2.0)	.070
Type of surgery			.4
Decompression alone	54 (35.1%)	24 (26.7%)	
Decompression with fusion	96 (62.3%)	63 (70.0%)	
Instrumented fusion alone	<6 (<4%)	<6 (<6%)	
Surgical approach			.4
Anterior	<6 (<4%)	<6 (<6%)	
Combined	12 (7.3%)	<6 (<6%)	
Posterior	135 (82.3%)	81 (80.2%)	
Year of treatment			.011
2019	97 (59.1%)	76 (75.2%)	
2020	67 (40.9%)	25 (24.8%)	
Follow-up (days)	263.7 (219.4)	249.7 (198.4)	.6
Home time (days)	442.3 (304.7)	293.4 (285.7)	<.001
Home time 3 months or less	41 (25.0%)	39 (38.6%)	.027
Died	70 (42.7%)	72 (71.3%)	<.001

Abbreviations: ICU, intensive care unit; ED, Emergency Department.

a n (%) for categorical variables; Mean (SD) for continuous variables.

b Pearson’s Chi-squared test; Welch two sample *t*-test.

**Figure 2.**
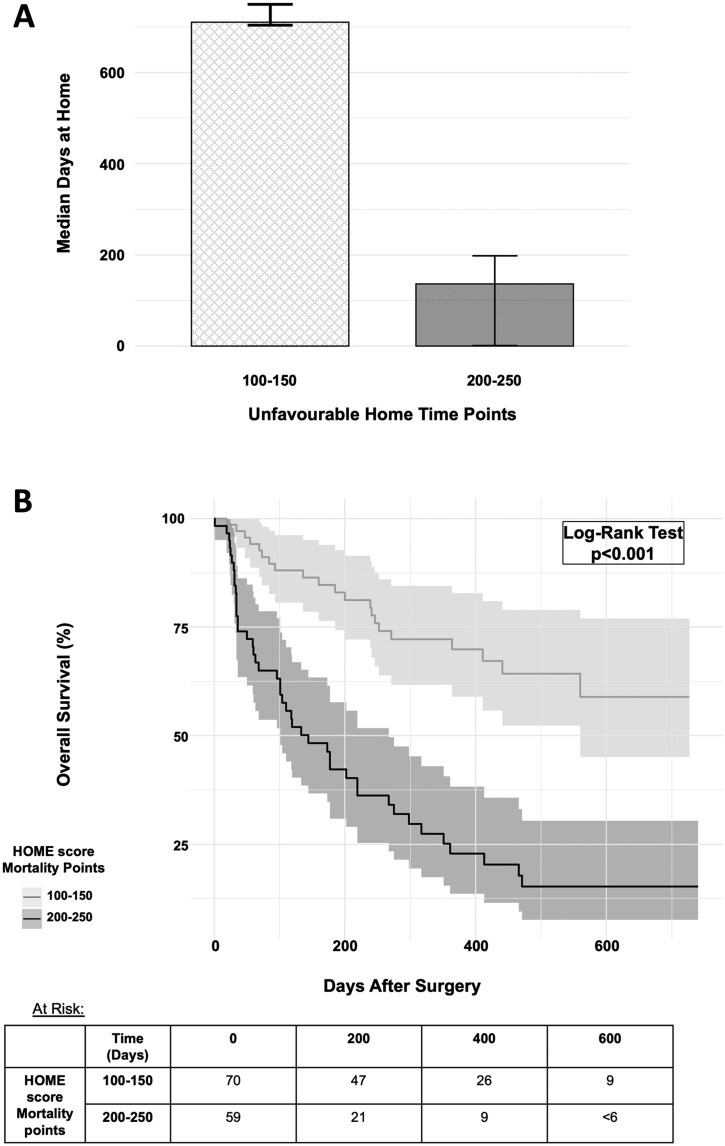
(A) Comparisons of median home time among hold-out test patients with unfavorable home time points between 100 and 150 compared to 200 and 250 points on the Home time and Overall survival after Metastatic spine tumor surgery Estimator (HOME score) nomogram. (B) Comparison of overall survival among hold-out test patients with mortality points between 100 and 150 compared to 200 and 250 on the HOME score nomogram.

When evaluating HOME scores on the hold-out test cohort, the range of unfavorable home time points was 54–289 points (interquartile range: 127–195 points), and the range of mortality points was 67-294 points (interquartile range: 139-211 points). Patients with lower unfavorable home time points (*N* = 82, total unfavorable home time points 100-150) had significantly more median days at home (710 days 95% CI: 704-750 vs 136 days 95% CI: 1-198 days, *P* < .001 t-test) than patients with greater unfavorable home time points (*N* = 50, total unfavorable home time points 200-250, Figure 2A). Similarly, patients with more mortality points (*N* = 59, total mortality points 200-250) had significantly shorter survival (*P* < .001 log-rank test) than patients with fewer mortality points (*N* = 70, total mortality points 100-150, Figure 2B). Conversely, patients within the lowest home time quartile had significantly greater unfavorable home time points on the HOME score compared to patients within the highest home time quartile (195 points [95% CI: 183-208 points] vs 141 points [95% CI: 133-154 points, *P* < .001). As well, patients within the lowest survival quartile had significantly greater HOME score mortality points compared to patients within the highest survival quartile (202 points [95% CI: 189-215 points] vs 154 point [95% CI: 133-172 points], *P* < .001). In sum, patients with greater unfavorable home time points, and mortality points had significantly fewer days at home, and shorter survival, respectively, and patients with shorter home time and survival, were found to have significantly greater unfavorable home time points and mortality points, respectively (Figure 2).

**Figure 3.**
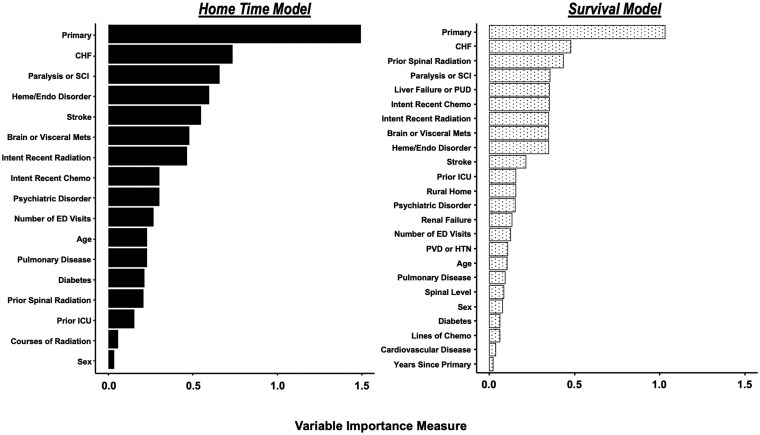
Variable importance of covariates quantified through standardized coefficients of the home time and survival models.

Ranking of standardized coefficients found the top 3 variables contributing to postoperative home time prediction were primary cancer origin, presence of CHF, and presence of paralysis or SCI (Figure 3). Similarly, the top 3 contributing variables for survival prediction were primary cancer origin, presence of CHF, and history of prior spinal radiation, followed closely by presence of paralysis or SCI. These variables were also found to be associated with significant differences in patient survival curves (Supplementary Figure 4—see online supplementary material for a color version of this figure).

## Discussion

In this study, we present the development and validation of a clinical prediction model for unfavorable home time and survival termed the HOME score. The nomogram specifying the trained and validated model is freely available, and readily transportable for further prospective validation and eventual clinical application. Discriminative and accuracy performance metrics were comparable to prior prognostic models for spine metastases.^11^ Importantly there was no significant loss in model performance after cross-validation or hold-out testing on a temporally distinct test cohort that differed significantly from the training cohort with respect to comorbidities, rates of various primary cancers, and systemic treatments offered prior to surgery. This work is a significant advancement for tailored patient centered pre-operative assessment and counseling for patients with this advanced form of cancer. Moreover, the HOME score’s ease of use is related to the inclusion of variables readily available to a primary care practitioner within any hospital setting.

### Comparison to Prior Models

There are limited prognostic models available for predicting home time after surgery. A recent study by Hallet et al. describes the STAYHOME tool for predicting the likelihood of postoperative admission to a nursing home after surgery.^45^ This model demonstrated similar performance to the HOME score and was not validated for use among patients with metastatic cancer. One prior prediction model developed by Nater et al. integrated 1-year survival with likelihood of achieving the minimal clinically meaningful improvement in the EuroQol 5-dimension health-related quality of life score at 3-months as co-primary outcomes.^46^ This prior model was found to have a validated C-index of 0.69 for survival prediction, and 0.74 for the quality-of-life outcome. The HOME score had comparable performance on cross-validation and hold-out testing. Moreover, our model builds upon prior tools by integrating home time and survival as prediction endpoints. Home time is a pragmatic patient-centered outcome measure that quantifies a meaningful and easily understood outcome for patients.^21–23^^,^^47^ As a clinical endpoint it can be readily quantified using administrative population health data, which can facilitate future training or validation of this model in most health care settings. In contrast, conventional quality of life outcomes requires individual patient contact and self-reported questionnaires, which can hinder the measurement and collation of data for training, testing, or deployment of these models.

Outside of home time and quality of life, there have been numerous prognostic models for predicting overall survival after diagnosis of spine metastases. These include the Tokuhashi,^19^^,^^48^ Bauer,^18^ Tomita,^20^ Katagiri, van der Linden,^12–14^,^17^ Oswestry Spinal Risk Index,^16^ New England Spine Metastasis score,^15^ and the Skeletal Oncology Research Group (SORG) models. A recent systematic review compared the performance of these 8 models with respect to predicting 1-year survival. The AUC (95% CI) for predicting overall survival at 1 year of the Classic SORG, SORG Nomogram, Original Tokuhashi, Revised Tokuhashi, Tomita, Original Bauer, Modified Bauer, Katagiri, and Linden models were 0.77 (95% CI: 0.70-0.84), 0.78 (95% CI: 0.71-0.85), 0.78 (95% CI: 0.71-0.85), 0.77 (95% CI: 0.70-0.84), 0.70 (95% CI: 0.62-0.78), 0.71 (95% CI: 0.64-0.79), 0.78 (95% CI: 0.72-0.85), and 0.71 (95% CI: 0.63-0.78). In our study the HOME score predicted 1-year survival with an AUC of 0.76, which is comparable to prior models.

### Clinical Applications and Future Directions

Application of the HOME score to clinical settings is facilitated by the selective inclusion of predictors that are readily available to any health care providers and specification with an easily transportable nomogram. Assessment of each variable in our model can be ascertained from a patient’s treatment and health history and does not require specialized testing or expert domain knowledge. This can assist with transparency and communication between various providers and patients. This broad accessibility ensures that the model can be used in a wide range of clinical settings, potentially empowering patients and their families to engage in more informed discussions about expected outcomes and care planning.

Looking ahead, there are several avenues for future research. Prospective validation with clinical records outside of Ontario would be instrumental to ensuring accuracy of the model. Alternatively, the model can be validated on existent international registries, such as the metastatic tumor research outcome network.^49^ Moreover, incorporating additional ensemble predictors such as molecular profiling, patient pre-operative performance status, and imaging features could further refine the model’s predictive accuracy. These variables capture important nuances such as tumor treatment responsiveness, patient frailty, degree of neurologic compression, and spinal stability that are not currently reflected in administrative health data but are likely to inform prognostication. The inclusion of molecular data will likely contribute the most significant advances to model performance, as HOME score evaluation demonstrated that primary tumor biology was the most important variable contributing to the prediction estimate. Similarly, we also found our model was more prone to misclassify cases of lung cancer, relative to other cancers. This may be because of the lack of inclusion of molecular profiling, which can identify patients with targetable mutations that dramatically alter expected survival, and by extension, home time.

### Strengths and Limitations

Strengths of this study include reporting of an easily interpretable pragmatic model developed from a large and diverse patient sample drawn from multiple socioeconomic settings and pan-provincial treatment environments. These factors would be expected to dramatically improve generalizability and applicability of the HOME score. Our analysis also demonstrated robust model performance on training and testing, despite significant differences between the 2 cohorts. This resilience to variability underscores its potential utility across different clinical environments and patient populations, mitigating the referral bias that often limits the applicability of other prognostic models.

Limitations of the HOME score are those inherent to training on administrative health data. Prior validation studies of administrative health codes used to classify patient comorbidities, reported positive predictive values for case definitions that ranged from 60% to 85%.^42^ This likely imposes a ceiling effect to model performance, necessitating future studies with prospectively collected data as mentioned above. HOME score performance and calibration were also limited by the class imbalance for the unfavorable home time outcome. Future harmonized dataset can provide more training cases of patients with postsurgical home time of 3 months or less, to more accurately calibrate predictions. The model was also limited by the lack of availability of patient performance measures, such as the Eastern Cooperative Oncology Group and Karnofsky performance measures, imaging finings, pain assessments, nutritional status, tumor molecular profiles, and patient serum albumin levels, which have all been found to prognosticate outcomes for patients undergoing surgery for a spine metastasis.^50^ A further limitation relates to the HOME score being derived and validated exclusively with surgically treated patients. Accordingly, its applicability to nonsurgical populations should be interpreted with caution. Nonoperative patients represent a heterogeneous group with differing clinical and personal factors influencing treatment selection, some of which were not captured in this study, including patient preferences, extent of epidural compression, and radiographic features. Lastly, advancements in early detection of cancer, minimally invasive surgery, adjuvant radiation, and systemic therapy will likely render the HOME score less reliable in the future. Accordingly ongoing retraining of the model with contemporary population health data will likely be an ongoing requirement to maintain accuracy of HOME score predictions. Moreover, generating novel data linkages between populatio7n health data, with prospectively collected assessments of performance status, patient reported quality of life, and granular data on tumor molecular profiles is an important next step to prospectively validate the HOME score, and potentially improve the performance of the model in a subsequent version.

## Conclusion

We have produced a new score that accurately predicts home time and survival for patients undergoing surgery for spinal metastases. The HOME score and it’s predicted outcomes are easily interpretable and specified through a readily available nomogram. This represents a significant advancement which will undoubtedly help to facilitate improvements in patient centered pre-operative counseling and treatment decision making. Future prospective validation and integration with molecular, functional, imaging data are next steps to improving performance.

## Supplementary Material

vdag010_Supplementary_Data

## Data Availability

The dataset from this study is held securely in coded form at the Institute for Clinical Evaluative Sciences (ICES). While data sharing agreements prohibit ICES from making the dataset publicly available, access may be granted to those who meet prespecified criteria for confidential access, available at www.ices.on.ca/DAS. The full dataset creation plan and underlying analytic code are available from the authors upon request, understanding that the computer programs may rely upon coding templates or macros that are unique to ICES and are therefore either inaccessible or may require modification.

## References

[1] Shakil H , Malhotra AK, Badhiwala JH, et al. Contemporary trends in the incidence and timing of spinal metastases: a population-based study. Neurooncol Adv. 2024;6:vdae051. 10.1093/NOAJNL/VDAE051 38680988 PMC11046986

[2] Versteeg AL , Sahgal A, Rhines LD, AOSpine Knowledge Forum Tumor, et al. Health related quality of life outcomes following surgery and/or radiation for patients with potentially unstable spinal metastases. The Spine Journal. 2021;21:492-499. 10.1016/J.SPINEE.2020.10.017 33098985

[3] Shakil H , Essa A, Malhotra AK, et al. Perioperative outcomes after minimally invasive and open surgery for treatment of spine metastases: a systematic review and meta-analysis. J Neurosurg Spine. 2024;42:215-229. 10.3171/2024.7.SPINE24518 39612501

[4] Nater A , Sahgal A, Fehlings M. Management—spinal metastases. Handb Clin Neurol. 2018;149:239-255. 10.1016/B978-0-12-811161-1.00016-5 29307356

[5] Laufer I , Rubin DG, Lis E, et al. The NOMS framework: approach to the treatment of spinal metastatic tumors. Oncologist. 2013;18:744-751. 10.1634/THEONCOLOGIST.2012-0293 23709750 PMC4063402

[6] Thio QCBS , Paulino Pereira NR, van Wulfften Palthe O, Sciubba DM, Bramer JAM, Schwab JH. Estimating survival and choosing treatment for spinal metastases: do spine surgeons agree with each other? J Orthop. 2021;28:134-139. 10.1016/j.jor.2021.11.015 34924728 PMC8665269

[7] Parkes CM. Accuracy of predictions of survival in later stages of cancer. Br Med J. 1972;2:29-31. 10.1136/BMJ.2.5804.29 4111472 PMC1789062

[8] Chow E , Harth T, Hruby G, Finkelstein J, Wu J, Danjoux C. How accurate are physicians’ clinical predictions of survival and the available prognostic tools in estimating survival times in terminally ill cancer patients? A systematic review. Clin Oncol (R Coll Radiol). 2001;13:209-218. 10.1053/CLON.2001.9256 11527298

[9] Chow E , Davis L, Panzarella T, et al. Accuracy of survival prediction by palliative radiation oncologists. Int J Radiat Oncol Biol Phys. 2005;61:870-873. 10.1016/J.IJROBP.2004.07.697 15708268

[10] Karhade AV , Shin JH, Schwab JH. Prognostic models for spinal metastatic disease: evolution of methodologies, limitations, and future opportunities. Ann Transl Med. 2019;7:219. 10.21037/ATM.2019.04.87 31297384 PMC6595198

[11] Ahmed AK , Goodwin CR, Heravi A, et al. Predicting survival for metastatic spine disease: a comparison of nine scoring systems. Spine J. 2018;18:1804-1814. 10.1016/J.SPINEE.2018.03.011 29567516

[12] Karhade AV , Thio QCBS, Ogink PT, et al. Predicting 90-day and 1-year mortality in spinal metastatic disease: development and internal validation. Neurosurgery. 2019;85:E671-E681. 10.1093/NEUROS/NYZ070 30869143

[13] Pereira NRP , Janssen SJ, Van Dijk E, et al. Development of a prognostic survival algorithm for patients with metastatic spine disease. J Bone Joint Surg Am. 2016;98:1767-1776. 10.2106/JBJS.15.00975 27807108

[14] Paulino Pereira NR , Mclaughlin L, Janssen SJ, et al. The SORG nomogram accurately predicts 3- and 12-months survival for operable spine metastatic disease: external validation. J Surg Oncol. 2017;115:1019-1027. 10.1002/JSO.24620 28346699

[15] Schoenfeld AJ , Le HV, Marjoua Y, et al. Assessing the utility of a clinical prediction score regarding 30-day morbidity and mortality following metastatic spinal surgery: the new England spinal metastasis score (NESMS). Spine J. 2016;16:482-490. 10.1016/J.SPINEE.2015.09.043 26409416

[16] Balain B , Jaiswal A, Trivedi JM, Eisenstein SM, Kuiper JH, Jaffray DC. The oswestry risk index: an aid in the treatment of metastatic disease of the spine. Bone Joint J. 2013;95-B:210-216. 10.1302/0301-620X.95B2.29323 23365031

[17] Van Der Linden YM , Dijkstra SPDS, Vonk EJA, Marijnen CAM, Leer JWH, Dutch Bone Metastasis Study Group Prediction of survival in patients with metastases in the spinal column: results based on a randomized trial of radiotherapy. Cancer. 2005;103:320-328. 10.1002/CNCR.20756 15593360

[18] Bauer HCF , Wedin R. Survival after surgery for spinal and extremity metastases. Prognostication in 241 patients. Acta Orthop Scand. 1995;66:143-146. 10.3109/17453679508995508 7740944

[19] Tokuhashi Y , Matsuzaki H, Oda H, Oshima M, Ryu J. A revised scoring system for preoperative evaluation of metastatic spine tumor prognosis. Spine (Phila Pa 1976). 2005;30:2186-2191. 10.1097/01.BRS.0000180401.06919.A5 16205345

[20] Tomita K , Kawahara N, Kobayashi T, Yoshida A, Murakami H, Akamaru T. Surgical strategy for spinal metastases. Spine (Phila Pa 1976. *)* 2001;26:298-306. 10.1097/00007632-200102010-00016 11224867

[21] Chesney TR , Haas B, Coburn NG, Recovery After Surgical Therapy for Older Adults Research–Cancer (RESTORE-Cancer) Group, et al. Patient-Centered time-at-home outcomes in older adults after surgical cancer treatment. JAMA Surg. 2020;155:e203754. 10.1001/JAMASURG.2020.3754 33026417 PMC7542525

[22] Arya S , Langston AH, Chen R, et al. Perspectives on home time and its association with quality of life after inpatient surgery among US veterans. JAMA Netw Open. 2022;5:E2140196. 10.1001/JAMANETWORKOPEN.2021.40196 35015066 PMC8753502

[23] Groff AC , Colla CH, Lee TH. Days spent at home–a patient-centered goal and outcome. N Engl J Med. 2016;375:1610-1612. 10.1056/NEJMP1607206/SUPPL_FILE/NEJMP1607206_DISCLOSURES.PDF 27783911 PMC5996758

[24] Shakil H , Malhotra AK, Essa A, et al. Days at home after treatment of spinal metastases: measurement and validation of a novel patient centered outcome. J Neurooncol. 2025;173:739-749. 10.1007/S11060-025-05014-Z 40111577

[25] Collins GS , Moons KGM, Dhiman P, et al. TRIPOD+AI statement: updated guidance for reporting clinical prediction models that use regression or machine learning methods. BMJ. 2024;385:e078378. 10.1136/BMJ-2023-078378 38626948 PMC11019967

[26] Schull MJ , Azimaee M, Marra M, et al. ICES: data, discovery, better health. Int J Popul Data Sci. 2020;4:1135. 10.23889/IJPDS.V4I2.1135 32935037 PMC7477779

[27] Shakil H , Malhotra AK, Essa A, et al. Chordoma incidence, treatment, and survival in the 21st century: a population-based Ontario cohort study. J Neurosurg. 2024;142:702-711. 10.3171/2024.6.JNS24426 39423422

[28] Finkelstein JA , Zaveri G, Wai E, Vidmar M, Kreder H, Chow E. A population-based study of surgery for spinal metastases. Journal of Bone and Joint Surgery—Series B. 2003;85-B:1045-1050. 10.1302/0301-620X.85B7.14201/LETTERTOEDITOR 14516044

[29] Brastianos HC , Nguyen P, Sahgal A, Eisenhauer EA, Baetz T, Hanna TP. Association of innovations in radiotherapy and systemic treatments with clinical outcomes in patients with melanoma brain metastasis from 2007 to 2016. JAMA Netw Open. 2020;3:e208204. 10.1001/JAMANETWORKOPEN.2020.8204 32663310 PMC7339137

[30] Bhanot K , Widdifield J, Huang A, Paterson JM, Shultz DB, Finkelstein J. Survival after surgery for spinal metastases: a population-based study. Can J Surg. 2022;65:E512-E518. 10.1503/CJS.000921 35926885 PMC9363129

[31] Malhotra AK , Nathens AB, Shakil H, et al. Days at home after traumatic brain injury: moving beyond mortality to evaluate patient-centered outcomes using population health data. Neurology. 2024;103:e209904. 10.1212/WNL.0000000000209904/SUPPL_FILE/SUPPLEMENT.PDF 39284113

[32] McIsaac DI , Talarico R, Jerath A, Wijeysundera DN. Days alive and at home after hip fracture: a cross-sectional validation of a patient-centred outcome measure using routinely collected data. BMJ Qual Saf. 2023;32:546-556. 10.1136/BMJQS-2021-013150 PMC10447366 34330880

[33] Yu AYX , Fang J, Porter J, Austin PC, Smith EE, Kapral MK. Hospital-based cohort study to determine the association between home-time and disability after stroke by age, sex, stroke type and study year in Canada. BMJ Open. 2019;9:e031379. 10.1136/BMJOPEN-2019-031379 PMC6858198 31719083

[34] Jerath A , Austin PC, Wijeysundera DN. Days alive and out of hospital: validation of a patient-centered outcome for perioperative medicine. Anesthesiology. 2019;131:84-93. 10.1097/ALN.0000000000002701 31094760

[35] Patchell RA , Tibbs PA, Regine WF, et al. Direct decompressive surgical resection in the treatment of spinal cord compression caused by metastatic cancer: a randomised trial. Lancet. 2005;366:643-648. 10.1016/S0140-6736(05)66954-1 16112300

[36] Fehlings MG , Nater A, Tetreault L, et al. Survival and clinical outcomes in surgically treated patients with metastatic epidural spinal cord compression: Results of the prospective multicenter AOSpine study. J Clin Oncol. 2016;34:268-276. 10.1200/JCO.2015.61.9338 26598751

[37] Dea N , Versteeg AL, Sahgal A, et al. Metastatic spine disease: should patients with short life expectancy Be denied surgical care? An international retrospective cohort study. Neurosurgery. 2020;87:303-311. 10.1093/NEUROS/NYZ472 31690935 PMC7360875

[38] Shah AA , Schwab JH, Shah AA, Schwab JH. Predictive modeling for spinal metastatic disease. Diagnostics (Basel). 2024;14:962. 10.3390/DIAGNOSTICS14090962 *Vol 14Page 962* 38732376 PMC11083521

[39] MacLean MA , Touchette CJ, Georgiopoulos M, AO Spine Knowledge Forum Tumor, et al. Systemic considerations for the surgical treatment of spinal metastatic disease: a scoping literature review. Lancet Oncol. 2022;23:e321-e333. 10.1016/S1470-2045(22)00126-7 35772464 PMC9844540

[40] Lee TH , Marcantonio ER, Mangione CM, et al. Derivation and prospective validation of a simple index for prediction of cardiac risk of major noncardiac surgery. Circulation. 1999;100:1043-1049. 10.1161/01.CIR.100.10.1043 10477528

[41] Quan H , Sundararajan V, Halfon P, et al. Coding algorithms for defining comorbidities in ICD-9-CM and ICD-10 administrative data. Med Care. 2005;43:1130-1139. 10.1097/01.MLR.0000182534.19832.83 16224307

[42] Quan H , Li B, Duncan Saunders L, IMECCHI Investigators, et al. Assessing validity of ICD-9-CM and ICD-10 administrative data in recording clinical conditions in a unique dually coded database. Health Serv Res. 2008;43:1424-1441. 10.1111/J.1475-6773.2007.00822.X 18756617 PMC2517283

[43] van Ingen T , Matheson FI. The 2011 and 2016 iterations of the Ontario marginalization index: updates, consistency and a cross-sectional study of health outcome associations. Canadian Journal of Public Health. 2022;113:260-271. 10.17269/S41997-021-00552-1 34432255 PMC8975983

[44] Shakil H , Dea N, Malhotra AK, et al. Who gets better after surgery for degenerative cervical myelopathy? A responder analysis from the multicenter Canadian spine outcomes and research network. Spine J. 2025;25:276-289. 10.1016/J.SPINEE.2024.09.033 39424073

[45] Hallet J , Mahar AL, Chan WC, Recovery After Surgical Therapy for Older Adults Research—Cancer (RESTORE-Cancer) Group, et al. A predictive tool for ability to remain at home after cancer surgery in older adults. JAMA Surg. 2025;160:903-911. 10.1001/JAMASURG.2025.1888 40560594 PMC12199182

[46] Nater A , Chuang J, Liu K, et al. A personalized medicine approach for the management of spinal metastases with cord compression: development of a novel clinical prediction model for postoperative survival and quality of life. World Neurosurg. 2020;140:654-663.e13. 10.1016/J.WNEU.2020.03.098 32797992

[47] Bell M , Eriksson LI, Svensson T, et al. Days at home after surgery: an integrated and efficient outcome measure for clinical trials and quality assurance. EClinicalMedicine. 2019;11:18-26. 10.1016/J.ECLINM.2019.04.011 31317130 PMC6610780

[48] Tokuhashi Y , Matsuzaki H, Toriyama S, Kawano H, Ohsaka S. Scoring system for the preoperative evaluation of metastatic spine tumor prognosis. Spine (Phila Pa 1976). 1990;15:1110-1113. 10.1097/00007632-199011010-00005 1702559

[49] AO Spine Knowledge Forum Tumor. Metastatic Tumor Research and Outcomes Network (MTRON). Accessed February 16, 2025. https://clinicaltrials.gov/study/NCT02830451

[50] Rigney GH , Massaad E, Kiapour A, et al. Implication of nutritional status for adverse outcomes after surgery for metastatic spine tumors. J Neurosurg Spine. 2023;39:557-567. 10.3171/2023.5.SPINE2367 37439458

