# Supplementary material for: Development and validation of the Home time and Overall survival after Metastatic spine tumor surgery Estimator (HOME score)
Source: Neurooncol Adv. 2026 Jan 23;8(1):vdag010. doi: 10.1093/noajnl/vdag010 (PMC12932946; doi:10.1093/noajnl/vdag010)
Supplement: vdag010_Supplementary_Data [file vdag010_supplementary_data.docx]

SUPPLEMENTARY MATERIAL

Supplementary Methods:

*Data Preparation*

All data preparation and transformations were completed separately for the training and testing cohort to ensure no data leak. Age was provided within 5-year intervals due to ICES privacy and patient confidentiality requirements. Age categories were mapped to ordinal categories of 1 through 13 as reported in Supplementary Table 1. Age, sex, and home location were determined using the registered person’s database, with rural being defined as a community size less than 10,000 people based on Statistics Canada recommendations.^1^

Comorbidities were determined using validated codes.^2,3^ We grouped related comorbidities together based to ensure at least 6 cases (the minimum allowable in ICES) within the training and testing cohort. Diabetes included cases of complicated and uncomplicated diabetes. Cardiovascular disease included cases of arrythmia, myocardial infarction, or valvular disease. Pulmonary disease included cases of chronic obstructive pulmonary disease or pulmonary circulation disorders. Hematologic or endocrine disorders included cases of anemia, coagulopathy, fluid and electrolyte disorders, or hypothyroidism. Psychiatric disorders included cases of psychosis, depression, alcohol use disorder, drug use disorder.

Primary cancer groups were identified using International Classification of Diseases Oncology 3 Topography Codes that have been described in prior studies.^4–6^ Chemotherapy and radiation activities were recorded from the cancer centre reporting to Cancer Care Ontario through the Activity Level Reporting database (ALR). Systemic metastasis types and numbers were derived from the American Joint Committee on Cancer (AJCC) coding schemas within the Ontario Cancer Registry (OCR).^7^ The interval between cancer diagnosis and first treatment of spinal metastases was estimated by the time between the first record within the OCR and earliest treatment for spinal metastasis. Details pertaining to surgical treatment were gathered from claims within the Ontario Health Insurance Plan (OHIP) database. Entries pertaining to spine surgery linked to a neoplastic diagnosis code were used to determine details of the surgical approach and technique.

Neighborhood socioeconomic status was derived from the Ontario Marginalization database, using the marginalization index. Patients were grouped into ordinal socioeconomic groups based on the marginalization quintile of their home neighbourhood. A higher marginalization quintile corresponded to lower home neighbourhood socioeconomic status.^8^

*Model Training*

We individually trained and cross-validated five model architectures for predicting home time and survival. For home time we trained and cross-validated a linear logistic regression model, a non-linear logistic regression model, a linear least absolute shrinkage and selection operator (LASSO) reduced logistic regression model, a non-linear LASSO reduced logistic regression model, and an extreme gradient boosted (XGBoost) binary classifier. For survival models we trained and cross-validated a linear cox proportional hazard model (CPH), a non-linear CPH, a linear LASSO reduced CPH, a non-linear LASSO reduced CPH, and a gradient boosted survival model. We externally tested each model trained on the entire training cohort with the hold out test set. LASSO, XGoost, and gradient boosted survival models was implemented using the *glmnet* version 4.1-8, *xgboost* version 1.7.8.1, and *gbm* version 2.2.2 packages in R, respectively.

Non-linear models were distinguished from linear models by virtue of treatment of continuous variables, where in non-linear models they were modelled using natural splines with 4 knots at the 5^th^, 35^th^, 65^th^, and 95^th^ percentile of the given continuous variable similar to prior studies.^9^ The optimal LASSO regularization hyperparameter was selected by optimizing the area under the receiver operating characteristic curve (AUC) for home time models, and the concordance index (C-index) for survival models within a 10-fold cross-validation. For LASSO models, regularization was used to remove any variable with a shrunken coefficient when implemented with the optimal hyperparameter. For categorical variables, levels with shrunken coefficients were collapsed, and any non-linear term with more than 3 terms eliminated was converted back to a linear term.

Optimal hyperparameters for the XGBoost home time model, and gradient boosted survival model were selected through a grid search method. For the XGBoost home time model, the grid was defined using a maximum of boosted iterations of 50, 100, 200, and 500; a maximum depth of 3, 6, and 10; learning rate of 0.01, 0.1, 0.2, 0.3; minimum loss reduction of 0, 1 and 5; column subsample ratio of 0.5, 0.75, and 1; minimum child sum of instance weight of 1, 3, 5, 10; and training instance subsample ratio of 0.5, 0.75, and 1. The optimal hyperparameters after search and cross validation are reported in Supplementary Table 6. For the gradient boosted survival model the grid was defined with an interaction depth of 1, 5, and 10; a shrinkage parameter of 0.001, 0.01, and 0.1; and a minimum number of observations in terminal nodes of 5, 10, and 20. The optimal number of trees was selected through optimizing the C-index on a 10-fold cross validation.

*Model Evaluation*

Optimally designed models were subsequently evaluated within a separate 10-fold cross validation of the training cohort. Performance was evaluated using the AUC for home time models, and the C-index for survival models. The 95% CI of performance metrics within the test-fold of the cross-validation was used to compare model architectures. When confidence intervals were overlapping, we selected the model with the least number of predictors for parsimony. We additionally re-trained each model on the entire training set and externally tested it on the hold-out cohort in a separate session to avoid data leak. Hold-out testing of the home time model consisted of assessment of the AUC, Brier score, and calibration plot. Hold-out testing of the survival model consisted of assessment of the C-index, Brier score, time-dependent AUC and calibration testing. Time-dependent testing was completed at 6-months, 1-year, and 2-years using the Chambless and Diao estimator.^10^

As a secondary validation of the HOME score model, we applied nomogram scoring to patients within the hold-out test cohort. We subsequently compared the median days at home with 95% confidence intervals for patients with unfavourable home time points between 100-150 to patients with points between 200-250. Confidence intervals were estimated from a 1000 iteration bootstrap. Additionally, we compared Kaplan-Meier survival curves among patients with mortality points between 100-150 to patients with points between 200-250. Confidence intervals were log-based, and a log-rank test was used to compare curves. To provide additional confidence in the HOME score model, we compared the median calculated HOME score unfavourable home time points for patients within the lowest post-operative home time quartile, to patients within the highest quartile. Similarly, we compared HOME score mortality points for patients within the lowest post-operative survival quartile to patients within the highest post-operative survival quartiles. Scores were compared using bootstrapped 95% confidence intervals of the median score within a given quartile.

We evaluated model errors by assessing misclassified cases. Classifications from the model were assigned on the basis of a threshold probability corresponding to the maximum Youden’s index of the ROC curve, as described previously.^11,12^ This was done to balance sensitivity and specificity of the model (Supplementary Figure 5). The threshold probability was used to dichotomize predictions for unfavourable home time, and overall survival at 6-months. Patients who were misclassified for either outcome were compared to correctly classified patient using univariable comparisons with the Welch’s t-test for continuous variables and the Chi-squared test for binary and categorical variables.

Variable importance was assessed in the final home time and survival model using standardized coefficients as previously described.^13^ Subsequently, Kaplan-Meier survival curves were evaluated using the entire patient cohort, with separate curves plotted according to patient groupings from the top 3 variables identified for home time and survival prediction.

*Supplementary References*

1. du Plessis V, Beshiri R, Bollman RD, Clemenson H. Definitions of “Rural.” In: *Agriculture and Rural Working Paper Series Working Paper No. 61*. Statistics Canada; 2002:1-37.

2. Quan H, Li B, Duncan Saunders L, et al. Assessing validity of ICD-9-CM and ICD-10 administrative data in recording clinical conditions in a unique dually coded database. *Health Serv Res*. 2008;43(4):1424-1441. doi:10.1111/J.1475-6773.2007.00822.X

3. Quan H, Sundararajan V, Halfon P, et al. Coding algorithms for defining comorbidities in ICD-9-CM and ICD-10 administrative data. *Med Care*. 2005;43(11):1130-1139. doi:10.1097/01.MLR.0000182534.19832.83

4. Finkelstein JA, Zaveri G, Wai E, Vidmar M, Kreder H, Chow E. A population-based study of surgery for spinal metastases. *Journal of Bone and Joint Surgery - Series B*. 2003;85(7):1045-1050. doi:10.1302/0301-620X.85B7.14201/LETTERTOEDITOR

5. Bhanot K, Widdifield J, Huang A, Paterson JM, Shultz DB, Finkelstein J. Survival after surgery for spinal metastases: a population-based study. *Can J Surg*. 2022;65(4):E512-E518. doi:10.1503/CJS.000921

6. Shakil H, Malhotra AK, Badhiwala JH, et al. Contemporary trends in the incidence and timing of spinal metastases: A population-based study. *Neurooncol Adv*. 2024;6(1). doi:10.1093/NOAJNL/VDAE051

7. Edge SB, Compton CC. The American Joint Committee on Cancer: the 7th edition of the AJCC cancer staging manual and the future of TNM. *Ann Surg Oncol*. 2010;17(6):1471-1474. doi:10.1245/S10434-010-0985-4

8. Moin JS, Moineddin R, Upshur REG. Measuring the association between marginalization and multimorbidity in Ontario, Canada: A cross-sectional study. *J Comorb*. 2018;8(1):2235042X18814939. doi:10.1177/2235042X18814939

9. Shakil H, Malhotra AK, Jaffe RH, et al. Factors influencing withdrawal of life-supporting treatment in cervical spinal cord injury: a large multicenter observational cohort study. *Crit Care*. 2023;27(1):1-11. doi:10.1186/S13054-023-04725-X/TABLES/3

10. Chambless LE, Diao G. Estimation of time-dependent area under the ROC curve for long-term risk prediction. *Stat Med*. 2006;25(20):3474-3486. doi:10.1002/SIM.2299

11. Schisterman EF, Faraggi D, Reiser B, Hu J. Youden Index and the optimal threshold for markers with mass at zero. *Stat Med*. 2008;27(2):297. doi:10.1002/SIM.2993

12. Youden WJ. Index for rating diagnostic tests. *Cancer*. 1950;3(1):32-35. doi:10.1002/1097-0142(1950)3:1<32::AID-CNCR2820030106>3.0.CO;2-3

13. Shakil H, Dea N, Malhotra AK, et al. Who gets better after surgery for degenerative cervical myelopathy? A responder analysis from the multicenter Canadian Spine Outcomes and Research Network. *Spine J*. 2025;25(2). doi:10.1016/J.SPINEE.2024.09.033

Supplementary Figure 1: Flow diagram outlining cohort assembly


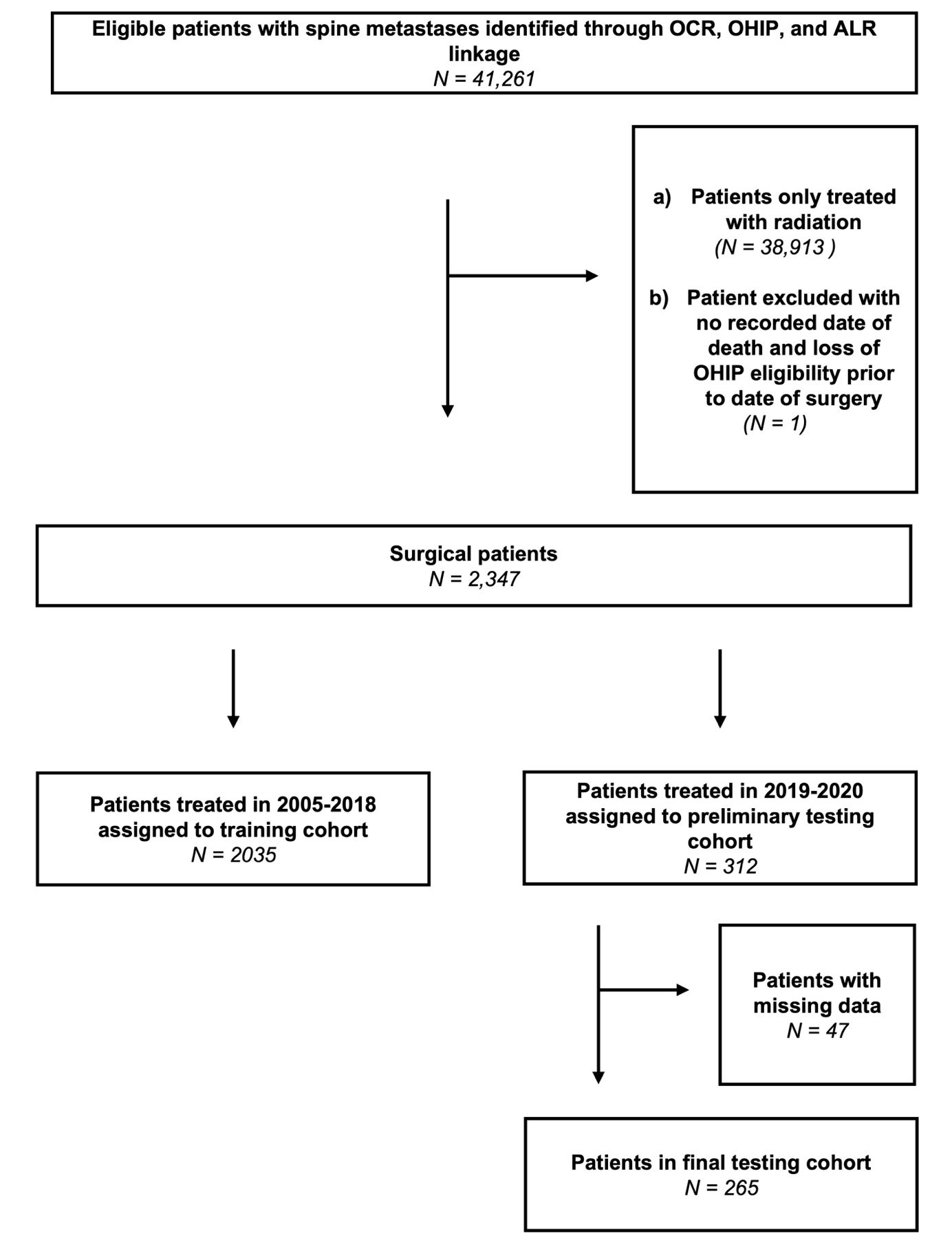


Supplementary Figure 2: Calibration plots of final integrated home time and survival model. The Brier score and area under the receiver operating characteristic curve (AUC) are provided for each endpoint.


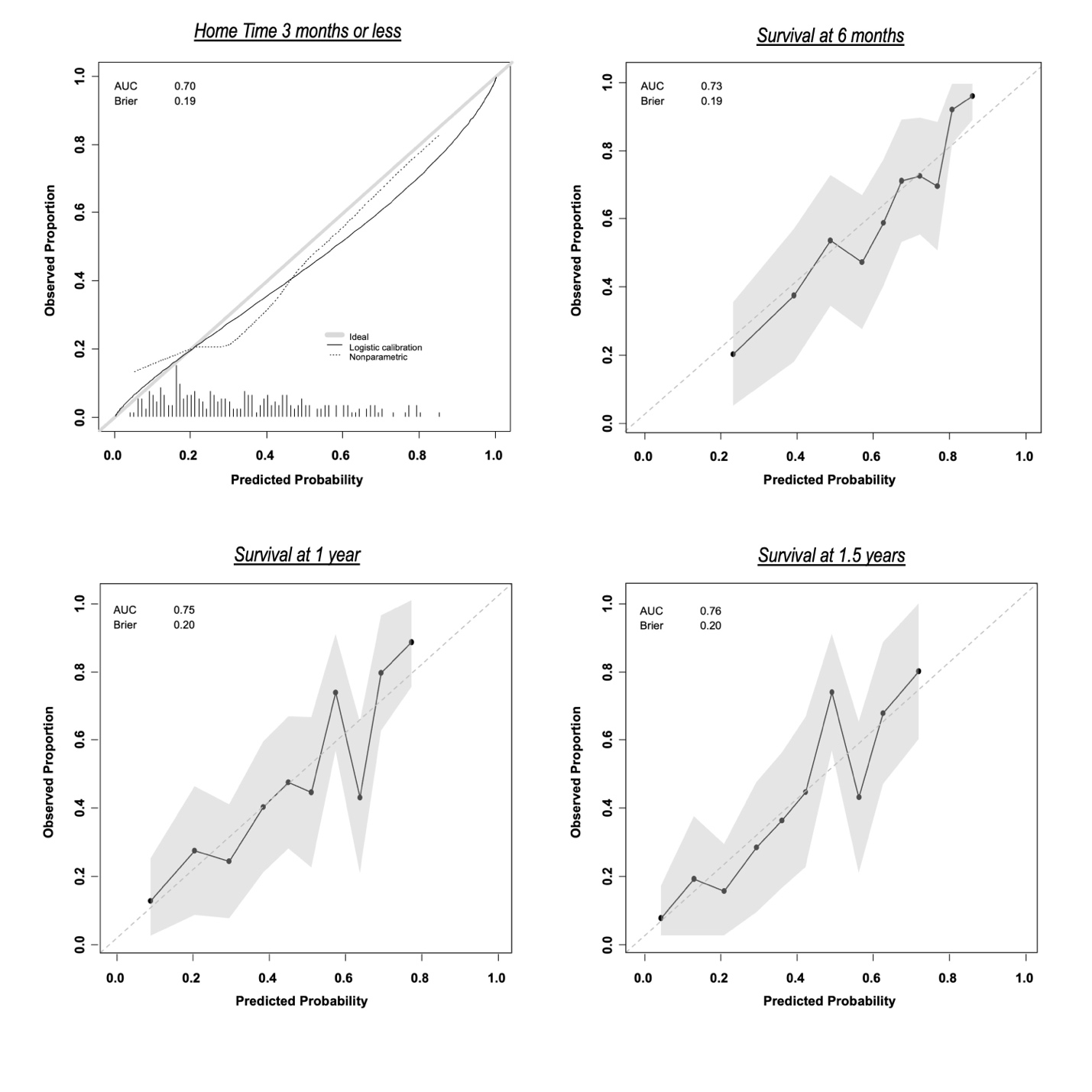


Supplementary Figure 3: Receiver operating characteristic curves for the Home time and Overall survival after Metastatic spine tumor surgery Estimator (HOME score) for predicting home time less than 3-months (left panel) and overall survival at 6-months (right panel). The optimal threshold probability based on Youden’s Index balances sensitivity and specificity.


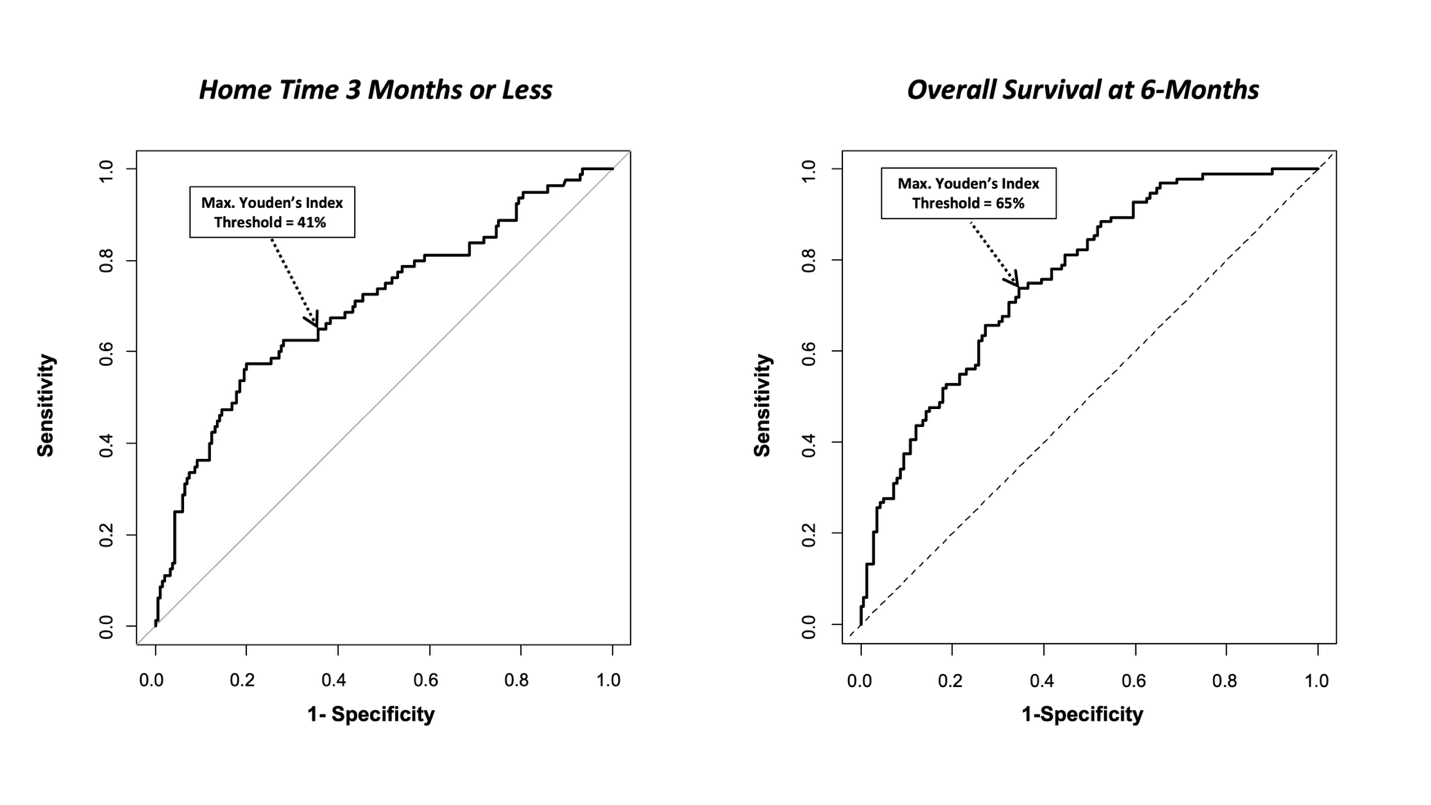


Supplementary Figure 4: Kaplan Meier survival curves for patients separated by variables with the highest importance for predictions of the Home time and Overall survival after Metastatic spine tumor surgery Estimator (HOME score).


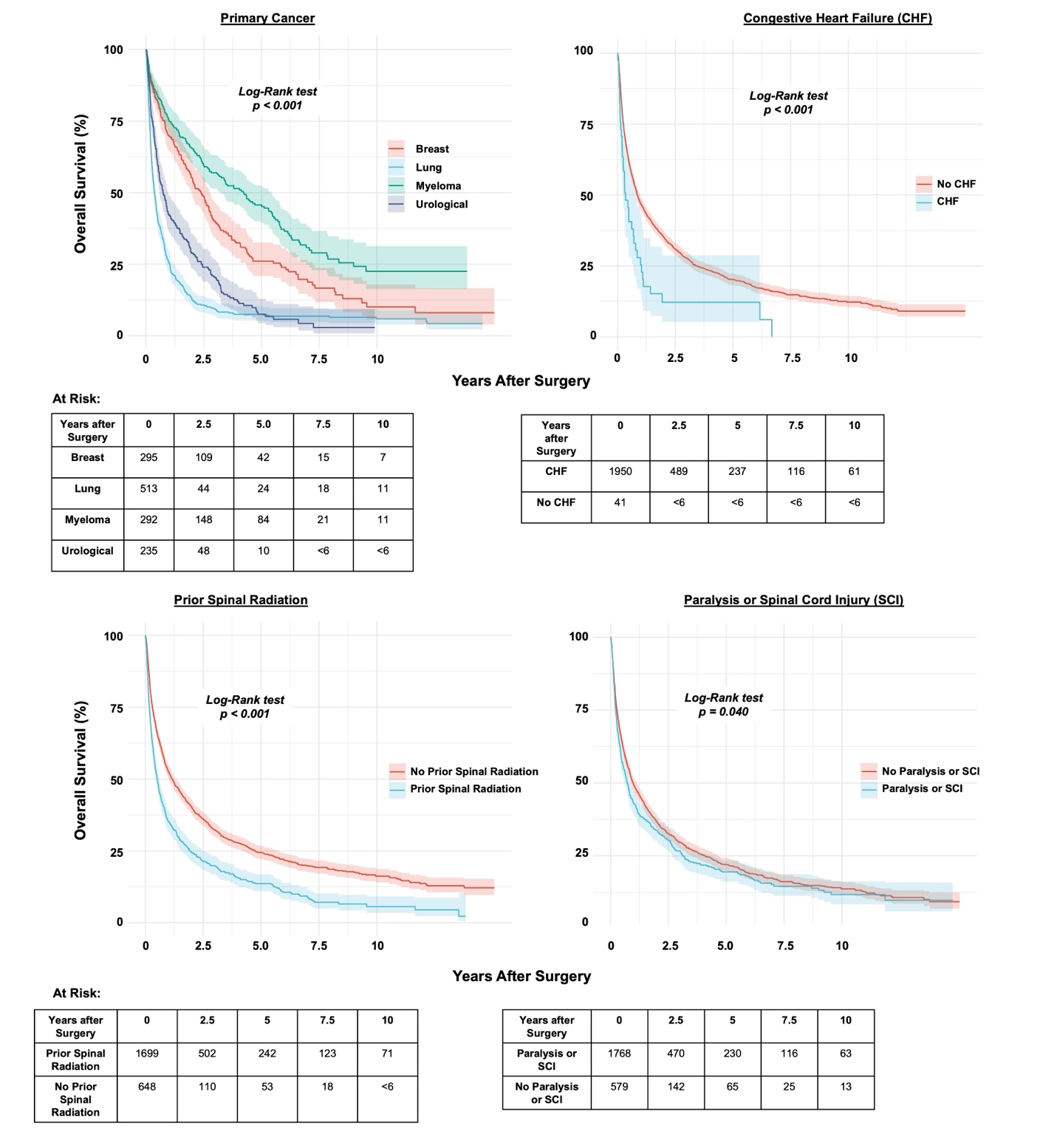


Supplementary Figure 5: Ten-fold cross validation of linear least absolute shrinkage selection operator (LASSO) models. The area under the receiver operating characteristic curve (AUC) of the logistic regression model predicting home time 3 months or less is plotted against the number of parameters included in the model (left panel). The concordance index (C-index) of the cox-proportional hazards model predicting overall survival is plotted against the number of parameters included in the model (right panel).


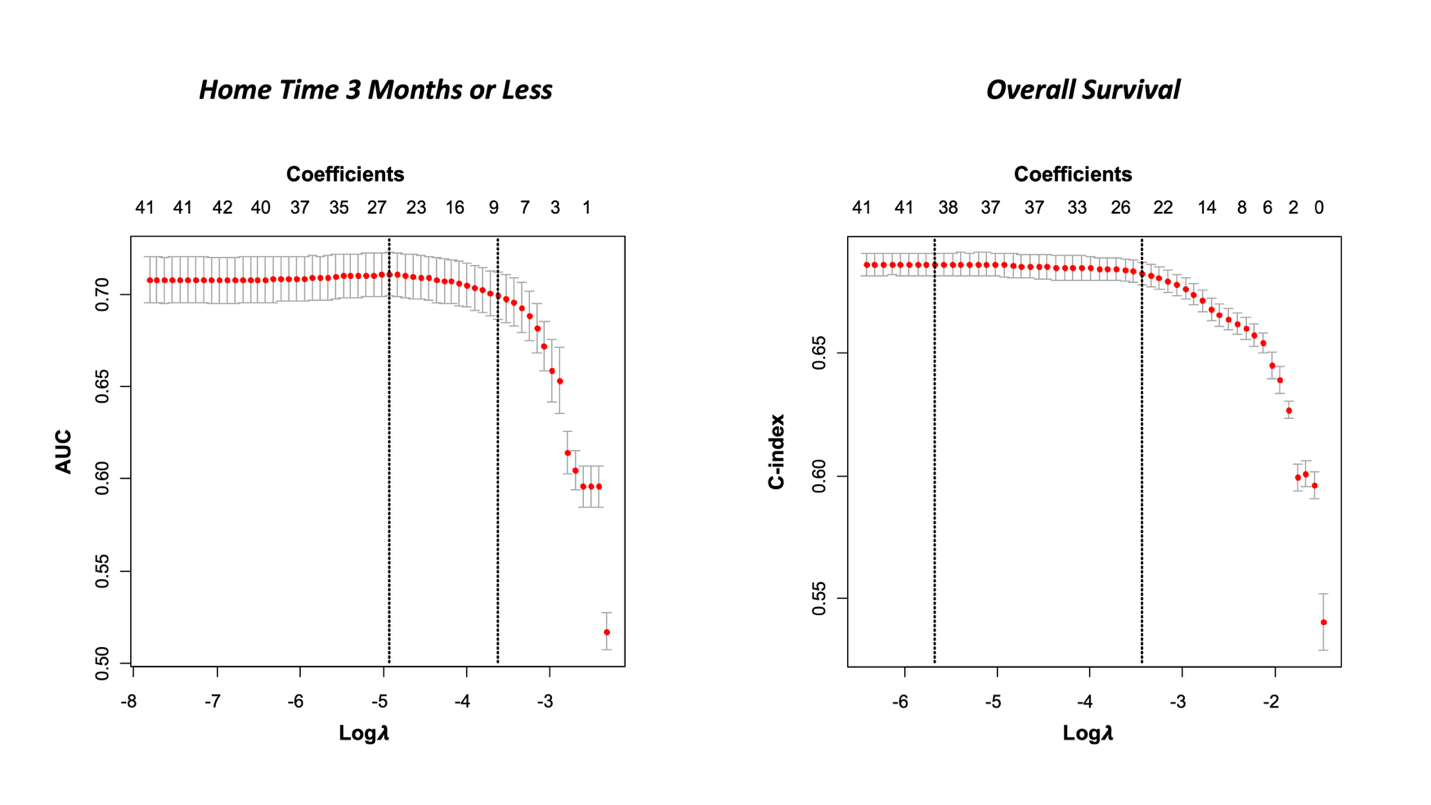


Supplementary Figure 6: Online web application for the **H**ome time and **O**verall survival after **M**etastatic spine surgery **E**stimator (HOME score). The online application can be accessed at (<https://shakilh.shinyapps.io/home_app/>). Users can input patient features and click the grey button to generate HOME score predictions for estimated probability of post-operative home time to be 3 months or less, along with overall survival probability at 6, 12, and 18 months after surgery. Predictions are provided along with 95% confidence intervals.


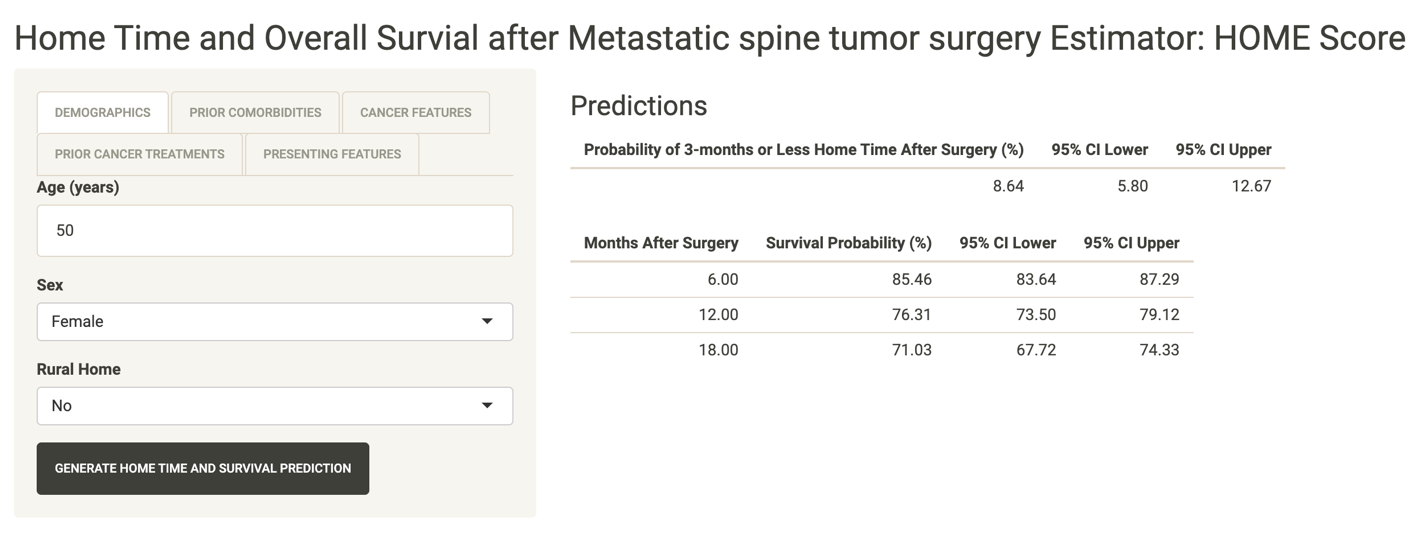


Supplementary Table 1: Data dictionary and sources for variables assessed in this study. Abbreviations: OCR, Ontario Cancer Registry, OHIP, Ontario Health Insurance Plan database, ALR, Cancer Care of Ontario Activity Level Reporting database; RPDB, Registered Person’s Database; CIHI, Canadian Institute for Health Information; DAD, Discharge Abstract Database; SDS, Same-day Surgeries Database, NACRS, National Ambulatory Care Reporting System; CCRS, the Continuing Care Reporting System; NRS, National Rehabilitation Reporting System; OMHRS, Ontario Mental Health Reporting System; HCD, and Home Care Database.

| Feature | Data Source | Identification | Time-Window | Coding in Model |
| --- | --- | --- | --- | --- |
| **Age** | RPDB | - | Time of SM | Continuous (years):  0 = 18-24 years  1 = 25-29 years  2 = 30-34 years  3 = 35-39 years  4 = 40-44 years  5 = 45-49 years  6 = 50-54 years  7 = 55-59 years  8 = 60-64 years  9 = 65-69 years  10 = 70-74 years  11 = 75-79 years  12 = 80-84 years  13 = 85+ years |
| **Sex** | RPDB | - | Time of SM | Binary:  0 = Female (ref)  1 = Male |
| **Home Location** | RPDB | Statistics Canada definition of rural = community population less than 10,000 people | Time of SM | Binary:  0 = non-rural (ref)  1 = rural |
| **Distance to Nearest Cancer Centre** | RPDB | ICES Macro | Time of SM | Continuous (Km) |
| **Congestive Heart Failure** | CIHI-DAD & NACRS | CHF ICD-10:  I09.9, I11.0, I13.0, I13.2, I25.5, I42.0, I42.5–  I42.9, I43.x, I50.x, P29.0 | Within 5-years prior to SM | Binary:  0 = no (ref)  1 = yes CHF |
| **Renal Failure** | CIHI-DAD & NACRS | Renal Failure ICD-10:  I12.0, I13.1, N18.x,N19.x, N25.0, Z49.0–Z49.2, Z94.0, Z99.2 | Within 5-years prior to SM | Binary:  0 = no (ref)  1 = yes renal failure |
| **Stroke** | CIHI-DAD & NACRS | Stroke ICD-10:  G45.x, G46.x, H34.0, I60.x–I69.x | Within 5-years prior to SM | Binary:  0 = no (ref)  1 = yes stroke |
| **Lung Disease** | CIHI-DAD & NACRS | Chronic Pulmonary Disease ICD-10:  I27.8, I27.9, J40.x–J47.x, J60.x–J67.x, J68.4, J70.1, J70.3  Pulmonary Circulation Disorders ICD-10:  I26.x, I27.x, I28.0, I28.8, I28.9 | Within 5-years prior to SM | Binary:  0 = no (ref)  1 = yes (chronic pulmonary disease or pulmonary circulation disorders) |
| **Peripheral Vascular Disease or Hypertension** | CIHI-DAD & NACRS | PVD ICD-10:  I70.x, I71.x, I73.1, I73.8, I73.9, I77.1, I79.0, I79.2, K55.1, K55.8, K55.9, Z95.8, Z95.9  HTN ICD-10:  I10.x I11.x–I13.x, I15.x | Within 5-years prior to SM | Binary:  0 = no (ref)  1 = yes (PVD or HTN) |
| **Liver Failure or Peptic Ulcer Disease** | CIHI-DAD & NACRS | Liver Disease ICD-10: B18.x, I85.x, I86.4, I98.2, K70.x, K71.1, K71.3–K71.5, K71.7, K72.x–K74.x, K76.0, K76.2–K76.9, Z94.4  PUD ICD-10:  K25.7, K25.9, K26.7, K26.9, K27.7, K27.9, K28.7, K28.9 | Within 5-years prior to SM | Binary:  0 = no (ref)  1 = yes (Liver disease or PUD) |
| **Hematologic or Endocrine Disorder** | CIHI-DAD & NACRS | Anemia ICD-10:  280.0, 648.2, 280.1–281.9, 285.2, 285.9  Coagulopathy ICD-10:  286.x, 287.1, 287.3–287.5  Hypothyroidism ICD-10:  243–244.2, 244.8, 244.9  Fluid & Electrolyte Disorders ICD-10:  E22.2, E86.x, E87.x | Within 5-years prior to SM | Binary:  0 = no (ref)  1 = yes (Anemia or Coagulopathy or Hypothyroidism or Fluid & Electrolyte Disorders) |
| **Diabetes** | CIHI-DAD & NACRS | Diabetes ICD-10:  E10.0, E10.1, E10.9, E11.0, E11.1, E11.9, E12.0, E12.1, E12.9, E13.0, E13.1, E13.9, E14.0, E14.1, E14.9, E10.2–E10.8, E11.2–E11.8, E12.2–E12.8, E13.2–E13.8, E14.2–E14.8 | Within 5-years prior to SM | Binary:  0 = no (ref)  1 = yes Diabetes |
| **Cardiovascular Disease** | CIHI-DAD & NACRS | MI ICD-10:  I21.x, I22.x, I25.2  Arrhythmia ICD-10:  I44.1–I44.3, I45.6, I45.9,  I47.x–I49.x, R00.0, R00.1, R00.8, T82.1, Z45.0, Z95.0  Valvular Disease ICD-10:  A52.0, I05.x–I08.x, I09.1,  I09.8, I34.x–I39.x, Q23.0–Q23.3, Z95.2–Z95.4 | Within 5-years prior to SM | Binary:  0 = no (ref)  1 = yes (MI or Arrhythmia or Valvular Disease) |
| **Psychiatric Disorder** | CIHI-DAD & NACRS | Alcohol Abuse ICD-10:  F10, E52, G62.1, I42.6, K29.2, K70.0, K70.3, K70.9, T51.x, Z50.2, Z71.4, Z72.1  Drug Abuse ICD-10:  F11.x–F16.x, F18.x, F19.x, Z71.5, Z72.2  Psychoses ICD-10:  F20.x, F22.x–F25.x, F28.x, F29.x, F30.2, F31.2, F31.5  Depression ICD-10:  F20.4, F31.3–F31.5, F32.x,  F33.x, F34.1, F41.2, F43.2 | Within 5-years prior to SM | Binary:  0 = no (ref)  1 = yes (Alcohol Abuse or Drug Abuse or Psychoses or Depression) |
| **Years Since Primary Cancer Diagnosis** | OCR | - | Lookback to inception of OCR (Jan 1964) | Continuous (years) |
| **Primary Cancer** | OCR | Breast ICD-O3:  C500-509  Prostate ICD-O3:  C619  Lung ICD-O3:  C340-349  GI ICD-O3:  C180-189, 260, 199, 209, 210-212,218, 150-179  Myeloma ICD-O3:  C421  Urological ICD-O3:  C649, 659, 670-689  Lymphoma ICD-O3:  C024, 098, 099, 111, 142, 379, 422, 770-779  Melanoma ICD-O3:  C440-449  Thyroid ICD-O3:  C739  Hepatobiliary ICD-O3:  C220, 221, 239, 240-259, 480-482, 268, 269, 488  Gynecological ICD-O3:  C54x, 539, 559, 569, 570-574, 577-579 | Lookback to inception of OCR (Jan 1964) | Categorical:  Breast (ref)  Prostate  Lung  GI  Myeloma  Urological  Lymphoma  Melanoma  Thyroid  Hepatobiliary  Gynecological  Other = none above |
| **Spine Level** | CIHI-DAD, OHIP, ALR | Cervical Decompression OHIP Fee Code:  Fee Code N500, N501, N569 with Neoplastic Dx code 140-239  Cervical Radiation Code:  SPCT, SPIC  Cervical CIHI-DAD Intervention location attribute:  CT, CX  Thoracolumbar Decompression OHIP:  Fee Code N502-508, N579, E362, N511, N512, N575 with Neoplastic Dx code 140-239  Thoracolumbar Radiation Code:  SPIL, SPIT, SPLS, SPTL  Thoracolumbar CIHI-DAD Intervention location attribute:  TH, TL, LB, LS | Within 1 year of surgery | Binary:  Cervical (ref) = yes (cervical decompression or cervical radiation, or cervical intervention location attribute)  Thoracolumbar = yes (thoracolumbar decompression or thoracolumbar radiation or thoracolumbar intervention location attribute) |
| **Number of Distant Metastases** | OCR | Single CS Mets at Dx AJCC 6 Code:  1-39  Multiple CS Mets at Dx AJCC 6 Code:  40-90 | Lookback to inception of OCR (Jan 1964) | Categorical:  None = (No CS Mets at Dx)  Single = Yes Single CS Mets at Dx  Multiple = Yes Multiple CS Mets at Dx |
| **Type of Systemic Metastases** | OCR | CS Mets Lung AJCC-6: 1  CS Mets Liver AJCC-6: 1  CS Mets Brain AJCC-6: 1 | Lookback to inception of OCR (Jan 1964) | Categorical:  None  Viseral = Yes (CS Mets Lung or CS Mets Liver)  Brain = Yes (CS Mets Brain)  Visceral and Brain = Yes (CS Mets Liver or CS Mets Lung) AND Yes (CS Mets Brain) |
| **Intent of Most Recent Chemotherapy** | ALR | ALR Systemic | Within 2 years prior to surgery | Categorical:  No prior Radiotherapy (ref)  Curative  Palliative |
| **Number of Lines of Chemotherapy** | ALR | ALR Systemic | Within 2 years prior to surgery | Continuous (counts) |
| **Intent of Most Non-Spine Radiotherapy** | ALR | ALR Radiation | Within 2 years prior to surgery | Categorical:  No prior Radiotherapy (ref)  Curative  Palliative |
| **Number of Courses of Radiotherapy** | ALR | ALR Radiation | Within 2 years prior to surgery | Continuous (counts) |
| **Prior Spinal Radiation** | ALR | Spinal Radiation Code:  COCC, SACR, SPCT, SPIC, SPIL SPIT, SPIW, SPLS, SPTL | Within 2 years prior to surgery | Binary:  0 = no (ref)  1 = yes spinal radiation |
| **Paralysis or Severe Spinal Cord Injury** | CIHI-DAD & NACRS & OHIP | Paralysis ICD-10:  G04.1, G11.4, G80.1, G80.2, G81.x, G82.x, G83.0–G83.4, G83.9  SCI OHIP:  Fee Code E383 with Neoplastic Dx code 140-239 | Within 5 years prior to surgery | Binary:  0 = no (ref)  1 = yes (Paralysis or SCI) |
| **Recent ICU Admission** | CIHI-DAD | ICU admission date with 1 year of date of surgery | Within 1 year prior to surgery | Binary:  0 = no (ref)  1 = yes ICU admission |
| **Number of ED Visits in 3 months Prior** | CIHI-NACRS | ED Registration date within 3-months of date of surgery | Within 3-months prior to surgery | Continuous (counts) |
| **Neighbourhood Socioeconomic Quintile** | ONMARG | Inverse of material deprivation quintile | 2-year prior to surgery | Categorical:  SES1 = material deprivation quintile 5 (ref)  SES 2 = material deprivation quintile 4  SES3 = material deprivation quintile 3  SES 4 = material deprivation quintile 2  SES 5 = material deprivation quintile 1 |
| **Surgery Type** | OHIP | Spinal Fusion OHIP:  Fee code E363, N516, E929, E924, R419, N517, N518, R303, N559, N580, E3569, E384, E371, E377, N528, N519, N514, N532, N515, E365, E362, E367, N539, R271, R350, E370, E382, E372, E376, N581, N540, E364, E366, N513, N582, E375, E548, E573, E574, R371, R459 with Neoplastic Dx code 140-239  Spinal Decompression OHIP:  Fee Code N500, N501, N569, N502-512, N579, E362, E360, E374, N574, N575, N520, E380, N524, N576, E361, E368, R264, E549, E565 with Neoplastic Dx code 140-239 | Time of Surgery | Categorical:  Decompression w/ Fusion = yes (Fusion AND  Decompression)  Decompression alone = yes (Decompression) AND No(Fusion)  Fusion alone = yes(Fusion) AND no(decompression) |
| **Surgical Approach** | OHIP | Anterior Spinal Surgery OHIP:  Fee code N500-N508, N569, N579, E362, E360, E363, N516, E929, N517, N518, R303, N559, N580, E365, E362, E367, N539, R271, R350 with Neoplastic Dx code 140-239  Posterior Spinal Surgery OHIP:  Fee code: E369-E377, E380, E384, E387, E567, E568, N509-N512, N514, N515, N519, N520, N524, N528, N532, N540, N574-N576, N581 | Time of Surgery | Categorical:  Combined = yes (Anterior) AND yes (Posterior)  Anterior = yes(Anterior)AND no(Posterior)  Posterior = yes(Posterior) AND no(Anterior) |
| **Home Time** | CIHI-DAD, SDS, NACRS, CCRS, NRS, OMHRS, HCD | ICES Macro: Days alive – days within healthcare institutions | 2-year follow-up from date of surgery | Continuous (days) |
| **Home Time 3 months or less** | - | - | - | Binary: 0 = Home Time > 90 days (ref)  1 = Home Time ≤ 90 days |
| **Survival** | RPDB | Time between surgery to earliest of death date, date of loss of OHIP eligibility, or Dec 31^st^ 2020 | Surgery to date of final follow-up | Time to event:  0 = censored  1 = death |

Supplementary Table 2: Summary of missing clinical records among patients assigned to the training and test cohort

|  | **Training**  **N = 2035** | **Preliminary Testing**  N = 312 |
| --- | --- | --- |
| **Age (years)** | 0 (0%) | 0 (0%) |
| **Sex** | 0 (0%) | 0 (0%) |
| **Rural** | <6 (<1%) | 0 (0%) |
| **Distance to Nearest Cancer Centre** | <6 (<1%) | 0 (0%) |
| **Congestive Heart Failure** | 315 (15.5%) | 41 (13.1%) |
| **Renal Failure** | 315 (15.5%) | 41 (13.1%) |
| **Stroke** | 0 (0%) | 0 (0%) |
| **Lung Disease** | 315 (15.5%) | 41 (13.1%) |
| **Peripheral Vascular Disease** | 315 (15.5%) | 41 (13.1%) |
| **Liver Failure or Peptic Ulcer Disease** | 315 (15.5%) | 41 (13.1%) |
| **Hematologic Disorder** | 315 (15.5%) | 41 (13.1%) |
| **Diabetes** | 315 (15.5%) | 41 (13.1%) |
| **Cardiovascular Disease** | 0 (0%) | 0 (0%) |
| **Psychiatric Disorder** | 315 (15.5%) | 41 (13.1%) |
| **Years Since Primary Cancer Diagnosis** | 0 (0%) | 0 (0%) |
| **Primary Cancer** | 0 (0%) | 0 (0%) |
| **Spine Level** | 48 (2.4%) | 6 (1.9%) |
| **Number of Distant Metastases** | <6 (<1%) | 0 (0%) |
| **Brain or Visceral Metastases** | 0 (0%) | 0 (0%) |
| **Intent of Most Recent Chemotherapy** | 0 (0%) | 0 (0%) |
| **Number of Lines of Chemotherapy** | 0 (0%) | 0 (0%) |
| **Intent of Most Non-Spine Radiotherapy** | 0 (0%) | 0 (0%) |
| **Number of Courses of Radiotherapy** | 0 (0%) | 0 (0%) |
| **Prior Spinal Radiation** | 0 (0%) | 0 (0%) |
| **Paralysis or Severe Spinal Cord Injury** | 0 (0%) | 0 (0%) |
| **Recent ICU Admission** | 0 (0%) | 0 (0%) |
| **Number of ED Visits in 3 months Prior** | 0 (0%) | 0 (0%) |

Supplementary Table 3: Comparison between clinical records of patients with and without missing data. Abbreviation: CHF, Congestive Heart Failure; PVD, Peripheral vascular disease; HTN, Hypertension; PUD, Peptic Ulcer Disease. GI, Gastrointestinal; HPB, Hepatobiliary. Gyne, Gynecological; SCI, Spinal Cord Injury; ICU, Intensive Care Unit; ED, Emergency Department; F/U, Follow-up. *^1^*n (%) for categorical variables; Mean (SD) for continuous variables. *^2^*Pearson’s Chi-squared test; Welch Two Sample t-test

|  | **Training Cohort** | | | **Testing Cohort** | | |
| --- | --- | --- | --- | --- | --- | --- |
|  | **No Missing Data**  N = 1,676*^1^* | **Missing Data**  N = 359*^1^* | **p-value***^2^* | **No Missing Data**  N = 265*^1^* | **Missing Data**  N = 47*^1^* | **p-value***^2^* |
| Age (years) |  |  | >0.9 |  |  | 0.8 |
| 20-59 | 649 (38.7%) | 140 (39.0%) |  | 78 (29.4%) | 16 (34.0%) |  |
| 60-79 | 927 (55.3%) | 198 (55.2%) |  | 166 (62.6%) | 25-30 (53-63%) |  |
| 80 or greater | 100 (6.0%) | 21 (5.8%) |  | 21 (7.9%) | <6 (<13%) |  |
| Sex |  |  | 0.8 |  |  | 0.8 |
| Female | 684 (40.8%) | 150 (41.8%) |  | 121 (45.7%) | 20 (42.6%) |  |
| Male | 992 (59.2%) | 209 (58.2%) |  | 144 (54.3%) | 27 (57.4%) |  |
| Rural Home | 206 (12.3%) | 42 (11.7%) | 0.8 | 33 (12.5%) | <6 (<13%) | >0.9 |
| Distance (Km) | 30.3 (47.2) | 29.1 (42.3) | 0.6 | 28.0 (51.7) | 34.4 (36.5) | 0.3 |
| CHF | 33 (2.0%) | <6 (<2%) | >0.9 | 7 (2.6%) | 0 (0.0%) | >0.9 |
| Renal | 38 (2.3%) | 0 (0.0%) | 0.6 | 6 (2.3%) | 0 (0.0%) | >0.9 |
| Stroke | 45 (2.7%) | <6 (<2%) | 0.12 | 15 (5.7%) | <6 (<13%) | 0.5 |
| Pulmonary | 148 (8.8%) | <6 (<2%) | 0.2 | 21 (7.9%) | <6 (<13%) | >0.9 |
| PVD or HTN | 423 (25.2%) | 7 (15.9%) | 0.2 | 64 (24.2%) | <6 (<13%) | >0.9 |
| Liver or PUD | 42 (2.5%) | <6 (<2%) | 0.2 | 11 (4.2%) | 0 (0.0%) | >0.9 |
| Heme/Endo | 241 (14.4%) | <6 (<2%) | 0.2 | 56 (21.1%) | <6 (<13%) | 0.2 |
| Diabetes | 217 (12.9%) | 8 (18.2%) | 0.4 | 42 (15.8%) | <6 (<13%) | 0.6 |
| Cardiac | 163 (9.7%) | <6 (<2%) | <0.001 | 39 (14.7%) | <6 (<13%) | 0.2 |
| Psychiatric | 81 (4.8%) | <6 (<2%) | 0.8 | 8 (3.0%) | 0 (0.0%) | >0.9 |
| Primary |  |  | 0.027 |  |  | 0.3 |
| Lung | 360 (21.5%) | 95 (26.5%) |  | 49 (18.5%) | 9 (19.1%) |  |
| Myeloma | 222 (13.2%) | 40 (11.1%) |  | 29 (10.9%) | <6 (<13%) |  |
| Breast | 193 (11.5%) | 48 (13.4%) |  | 45 (17.0%) | 9 (19.1%) |  |
| Other | 179 (10.7%) | 37 (10.3%) |  | 26 (9.8%) | <6 (<13%) |  |
| Prostate | 162 (9.7%) | 44 (12.3%) |  | 41 (15.5%) | 9 (19.1%) |  |
| Urological | 185 (11.0%) | 21 (5.8%) |  | 22 (8.3%) | 7 (14.9%) |  |
| GI | 123 (7.3%) | 18 (5.0%) |  | 22 (8.3%) | <6 (<13%) |  |
| Lymphoma | 81 (4.8%) | 24 (6.7%) |  | 8 (3.0%) | <6 (<13%) |  |
| HPB | 53 (3.2%) | 8 (2.2%) |  | <6 (<2%) | 0 (0.0%) |  |
| Melanoma | 48 (2.9%) | 13 (3.6%) |  | <6 (<2%) | <6 (<13%) |  |
| Gyne | 43 (2.6%) | 5-10 (2-3%) |  | 8 (3.0%) | <6 (<13%) |  |
| Thyroid | 27 (1.6%) | <6 (<2%) |  | <6 (<2%) | <6 (<13%) |  |
| Intent of Chemo |  |  | 0.8 |  |  | 0.027 |
| No Prior | 1,154 (68.9%) | 253 (70.5%) |  | 137 (51.7%) | 17 (36.2%) |  |
| Curative | 168 (10.0%) | 32 (8.9%) |  | 46 (17.4%) | 6 (12.8%) |  |
| Palliative | 354 (21.1%) | 74 (20.6%) |  | 82 (30.9%) | 24 (51.1%) |  |
| Lines of Chemo |  |  | 0.7 |  |  | 0.044 |
| 0 | 1,417 (84.5%) | 297 (82.7%) |  | 214 (80.8%) | 30-35 (64-74%) |  |
| 1 | 219 (13.1%) | 52 (14.5%) |  | 40 (15.1%) | 15 (31.9%) |  |
| 2 or greater | 40 (2.4%) | 10 (2.8%) |  | 10 (3.8%) | <6 (<13%) |  |
| Intent of Rads |  |  | 0.7 |  |  | 0.045 |
| No Prior | 1,210 (72.2%) | 251 (69.9%) |  | 158 (59.6%) | 21 (44.7%) |  |
| Curative | 241 (14.4%) | 55 (15.3%) |  | 62 (23.4%) | 11 (23.4%) |  |
| Palliative | 225 (13.4%) | 53 (14.8%) |  | 45 (17.0%) | 15 (31.9%) |  |
| Courses of Rads | 1.0 (4.9) | 1.6 (7.5) | 0.10 | 1.7 (6.9) | 4.4 (12.8) | 0.2 |
| No. of Mets |  |  | 0.003 |  |  | 0.5 |
| None | 1,395 (83.2%) | 269 (75.8%) |  | 259 (97.7%) | 46 (97.9%) |  |
| Single | 79 (4.7%) | 27 (7.6%) |  | <6 (<2%) | <6 (<13%) |  |
| Multiple | 202 (12.1%) | 59 (16.6%) |  | <6 (<2%) | 0 (0.0%) |  |
| Brain or Visceral |  |  | 0.8 |  |  | 0.6 |
| None | 1,559 (93.0%) | 334 (93.0%) |  | 259 (97.7%) | 47 (100.0%) |  |
| Visceral alone | 89 (5.3%) | 21 (5.8%) |  | <6 (<2%) | 0 (0.0%) |  |
| Brain alone | 17 (1.0%) | <6 (<2%) |  | 0 (0.0%) | 0 (0.0%) |  |
| Brain &Visceral | 11 (0.7%) | <6 (<2%) |  | <6 (<2%) | 0 (0.0%) |  |
| Spinal Level |  |  | >0.9 |  |  | 0.7 |
| Cervical | 514 (30.7%) | 94 (30.2%) |  | 77 (29.1%) | 10 (24.4%) |  |
| Thoracolumbar | 1,162 (69.3%) | 217 (69.8%) |  | 188 (70.9%) | 31 (75.6%) |  |
| Yrs Since Primary | 1.6 (2.5) | 1.7 (2.9) | 0.7 | 3.1 (4.1) | 3.4 (4.0) | 0.6 |
| Neoadjuvant. Rads | 428 (25.5%) | 140 (39.0%) | <0.001 | 56 (21.1%) | 24 (51.1%) | <0.001 |
| Paralysis or SCI | 434 (25.9%) | 67 (18.7%) | 0.005 | 71 (26.8%) | 7 (14.9%) | 0.12 |
| ICU Admission | 290 (17.3%) | 16 (4.5%) | <0.001 | 47 (17.7%) | <6 (<13%) | 0.082 |
| No. ED Visits | 2.0 (1.7) | 1.5 (1.6) | <0.001 | 2.1 (1.8) | 1.6 (2.0) | 0.081 |
| Post-op F/U | 772 (1,038) | 1,037 (1,235) | <0.001 | 258 (211) | 260 (233) | >0.9 |
| Died | 1,390 (82.9%) | 260 (72.4%) | <0.001 | 142 (53.6%) | 21 (44.7%) | 0.3 |
| Home Time (days) | 345.1 (290.0) | 414.3 (286.1) | <0.001 | 385.5 (305.7) | 456.0 (292.7) | 0.14 |
| Home Time 3 months or less | 542 (32.3%) | 83 (23.1%) | <0.001 | 80 (30.2%) | 11 (23.4%) | 0.4 |

Supplementary Table 4: Least absolute shrinkage and selection operator coefficients for optimal linear home time and survival models. Shrunken coefficients are reported with a “.”. Abbreviations: CHF, Congestive Heart Failure; PVD, Peripheral vascular disease; HTN, Hypertension; PUD, Peptic Ulcer Disease. GI, Gastrointestinal; HPB, Hepatobiliary. Gyne, Gynecological; SCI, Spinal Cord Injury; ICU, Intensive Care Unit; ED, Emergency Department.

|  | Home Time Model | Survival Model |
| --- | --- | --- |
| Age | 0.07 | 0.04 |
| Sex | 0.07 | 0.09 |
| Rural Home | . | 0.15 |
| Distance to Nearest Cancer Centre | . | . |
| CHF | 0.44 | 0.44 |
| Renal Failure | . | 0.12 |
| Stroke | 0.38 | 0.19 |
| Pulmonary Disease | 0.18 | 0.09 |
| PVD or HTN | . | -0.09 |
| Liver or PUD | . | 0.34 |
| Heme/Endo | 0.5 | 0.33 |
| Diabetes | -0.03 | -0.05 |
| Cardiac | . | -0.01 |
| Psychiatric Disorder | 0.16 | 0.13 |
| Years Since Primary | . | 0.01 |
| Brain or Visceral Mets: Visceral | 0.08 | 0.32 |
| Brain or Visceral Mets: Brain | . | -0.2 |
| Brain or Visceral Mets: Brain and Visceral | 0.14 | 0.3 |
| Primary: GI | 0.88 | 0.94 |
| Primary: Gyne | . | 0.26 |
| Primary: HPB | 0.65 | 0.82 |
| Primary: Lung | 1.07 | 0.94 |
| Primary: Lymphoma | -0.05 | -0.48 |
| Primary: Melanoma | 0.84 | 0.95 |
| Primary: Myeloma | -0.46 | -0.49 |
| Primary: Other | 0.09 | 0.18 |
| Primary: Prostate | . | 0.03 |
| Primary: Thyroid | -0.38 | -0.33 |
| Primary: Urological | 0.23 | 0.46 |
| Spinal Level | . | -0.08 |
| Number of Mets: Single | . | . |
| Number of Mets: Multiple | . | -0.01 |
| Intent of Chemo: Curative | -0.03 | -0.33 |
| Intent of Chemo: Palliative | -0.15 | . |
| Lines of Chemo | . | 0.11 |
| Intent of Rads: Curative | . | 0.13 |
| Intent of Rads: Palliative | 0.26 | 0.33 |
| Courses of Rads | 0.01 | . |
| Neoadjuvant Spinal Radiation | -0.12 | 0.43 |
| Paralysis or SCI | 0.57 | 0.34 |
| Prior ICU Admission | 0.09 | 0.15 |
| Number of ED Visits | 0.13 | 0.07 |

Supplementary Table 5: Least absolute shrinkage and selection operator coefficients for optimal non-linear home time and survival models. Shrunken coefficients are reported with a “.”. Abbreviations: CHF, Congestive Heart Failure; PVD, Peripheral vascular disease; HTN, Hypertension; PUD, Peptic Ulcer Disease. GI, Gastrointestinal; HPB, Hepatobiliary. Gyne, Gynecological; SCI, Spinal Cord Injury; ICU, Intensive Care Unit; ED, Emergency Department.

|  | Home Time Model | Survival Model |
| --- | --- | --- |
| Age: Spline 1 | . | -0.01 |
| Age: Spline 2 | . | . |
| Age: Spline 3 | 0.3 | 0.06 |
| Age: Spline 4 | 0..48 | 0.03 |
| Age: Spline 5 | 1.05 | 0.71 |
| Sex | 0.08 | 0.15 |
| Rural Home | . | 0.15 |
| Distance to Nearest Cancer Centre: Spline 1 | -0.2 | -0.14 |
| Distance to Nearest Cancer Centre: Spline 2 | . | . |
| Distance to Nearest Cancer Centre: Spline 3 | . | . |
| Distance to Nearest Cancer Centre: Spline 4 | . | . |
| Distance to Nearest Cancer Centre: Spline 5 | . | 0.14 |
| CHF | 0.46 | 0.4 |
| Renal Failure | . | 0.08 |
| Stroke | 0.35 | 0.14 |
| Pulmonary Disease | 0.18 | 0.07 |
| PVD or HTN | . | -0.09 |
| Liver or PUD | . | 0.26 |
| Heme/Endo | 0.42 | 0.25 |
| Diabetes | -0.02 | -0.06 |
| Cardiac | . | . |
| Psychiatric Disorder | 0.14 | 0.11 |
| Years Since Primary: Spline 1 | 0.4 | 0.09 |
| Years Since Primary: Spline 2 | . | 0.48 |
| Years Since Primary: Spline 3 | . | . |
| Years Since Primary: Spline 4 | . | -0.3 |
| Years Since Primary: Spline 5 | . | . |
| Brain or Visceral Mets: Visceral | 0.02 | 0.3 |
| Brain or Visceral Mets: Brain | . | -0.13 |
| Brain or Visceral Mets: Brain and Visceral | 0.15 | 0.29 |
| Primary: GI | 0.9 | 0.9 |
| Primary: Gyne | . | 0.26 |
| Primary: HPB | 0.65 | 0.83 |
| Primary: Lung | 1.13 | 1.04 |
| Primary: Lymphoma | -0.08 | -0.43 |
| Primary: Melanoma | 0.86 | 0.88 |
| Primary: Myeloma | -0.44 | -0.38 |
| Primary: Other | 0.04 | 0.19 |
| Primary: Prostate | . | . |
| Primary: Thyroid | -0.34 | -0.25 |
| Primary: Urological | 0.25 | 0.48 |
| Spinal Level | . | -0.05 |
| Number of Mets: Single | . | . |
| Number of Mets: Multiple | . | . |
| Intent of Chemo: Curative | -0.08 | -0.4 |
| Intent of Chemo: Palliative | -0.14 | -0.03 |
| Lines of Chemo: Spline 1 | . | . |
| Lines of Chemo: Spline 2 | . | -0.78 |
| Lines of Chemo: Spline 3 | . | . |
| Lines of Chemo: Spline 4 | . | . |
| Lines of Chemo: Spline 5 | . | . |
| Intent of Rads: Curative | . | -0.03 |
| Intent of Rads: Palliative | 0.22 | 0.07 |
| Courses of Rads: Spline 1 | -0.05 | -0.08 |
| Courses of Rads: Spline 2 | -0.38 | . |
| Courses of Rads: Spline 3 | . | 2.21 |
| Courses of Rads: Spline 4 | . | . |
| Courses of Rads: Spline 5 | 0.11 | . |
| Neoadjuvant Spinal Radiation | -0.15 | 0.39 |
| Paralysis or SCI | 0.54 | 0.32 |
| Prior ICU Admission | 0.02 | 0.08 |
| Number of ED Visits: Spline 1 | . | 0.1 |
| Number of ED Visits: Spline 2 | . | . |
| Number of ED Visits: Spline 3 | 0.51 | 0.47 |
| Number of ED Visits: Spline 4 | -0.88 | -0.37 |
| Number of ED Visits: Spline 5 | 0.85 | 0.29 |

Supplementary Table 6: Summary of hyperparameters used for cross-validated models.

|  | Hyperparameter | Home Time Model | Survival Model |
| --- | --- | --- | --- |
| LASSO-Linear | Regularization Parameter | 7.26 x 10^-3^ | 2.38 x 10^-3^ |
| LASSO-Non-Linear | Regularization Parameter | 7.26 x 10^-3^ | 2.17 x 10^-3^ |
| Gradient Boosted | Number of Trees | - | 35 |
|  | Interaction Depth | - | 10 |
|  | Shrinkage | - | 0.1 |
|  | Minimum Number of Observations in Terminal Nodes | - | 5 |
| Extreme Gradient Boosted | Boosted Iterations | 100 | - |
|  | Maximum Depth | 3 | - |
|  | Learning Rate | 0.1 | - |
|  | Minimum Loss Reduction | 5 | - |
|  | Column Subsample Ratio | 0.75 | - |
|  | Minimum Child Sum of Instance Weight | 10 | - |
|  | Training Instance Subsample Ratio | 0.5 | - |

Supplementary Table 7: Summary of final home time model for predicting home time of 3 months or less

|  | **Odds Ratio** | **95% CI** | **p-value** |
| --- | --- | --- | --- |
| Intercept | 0.09 | 0.02 – 0.32 | <0.001 |
| Age (per 5-year increase; ref 18-24 years) | 1.10 | 1.05 – 1.15 | <0.001 |
| Sex: Male vs Female | 1.04 | 0.81 – 1.33 | 0.766 |
| Rural Home | 2.08 | 0.97 – 4.48 | 0.061 |
| CHF | 1.73 | 0.92 – 3.26 | 0.089 |
| Pulmonary Disease | 1.26 | 0.87 – 1.84 | 0.227 |
| Heme/Endo | 1.82 | 1.33 – 2.48 | <0.001 |
| Diabetes | 0.81 | 0.58 – 1.13 | 0.206 |
| Psychiatric Disorder | 1.35 | 0.82 – 2.23 | 0.235 |
| Brain or Visceral Metastasis | 1.61 | 0.49 – 5.26 | 0.428 |
| Primary: GI vs Breast | 4.10 | 2.39 – 7.02 | <0.001 |
| Primary: Gyne vs Breast | 1.38 | 0.63 – 3.00 | 0.416 |
| Primary: HPB vs Breast | 3.40 | 1.73 – 6.66 | <0.001 |
| Primary: Lung vs Breast | 4.46 | 2.88 – 6.92 | <0.001 |
| Primary: Lymphoma vs Breast | 1.11 | 0.59 – 2.09 | 0.752 |
| Primary: Melanoma vs Breast | 4.21 | 2.17 – 8.18 | <0.001 |
| Primary: Myeloma vs Breast | 0.77 | 0.46 – 1.32 | 0.347 |
| Primary: Other vs Breast | 1.74 | 1.04 – 2.89 | 0.034 |
| Primary: Prostate vs Breast | 1.32 | 0.76 – 2.29 | 0.318 |
| Primary: Thyroid vs Breast | 0.55 | 0.15 – 1.97 | 0.359 |
| Primary: Urological vs Breast | 2.06 | 1.23 – 3.47 | 0.006 |
| Intent of Chemo: Curative vs no prior chemo | 0.75 | 0.50 – 1.11 | 0.153 |
| Intent of Chemo: Palliative vs no prior chemo | 0.74 | 0.55 – 0.99 | 0.046 |
| Intent of Rads: Curative vs no prior rads | 1.24 | 0.88 – 1.76 | 0.222 |
| Intent of Rads: Palliative vs no prior rads | 1.59 | 1.14 – 2.23 | 0.007 |
| Courses of Rads (per additional course; ref 0 course) | 1.01 | 0.99 – 1.03 | 0.255 |
| Neoadjuvant Spinal Radiation | 0.81 | 0.62 – 1.06 | 0.130 |
| Paralysis or SCI | 1.93 | 1.51 – 2.47 | <0.001 |
| Prior ICU Admission | 1.17 | 0.87 – 1.55 | 0.296 |
| Number of ED Visits (Per additional visit; ref 0 visits) | 1.18 | 1.10 – 1.25 | <0.001 |

Supplementary Table 8: Summary of final survival model predicting overall survival

|  | **Hazard Ratio** | **95% CI** | **p-value** |
| --- | --- | --- | --- |
| Age (per 5-year increase; ref 18-24 years) | 1.04 | 1.02 – 1.07 | **<0.001** |
| Sex: Male vs Female | 1.08 | 0.96 – 1.22 | 0.204 |
| Rural Home | 1.17 | 1.01 – 1.36 | **0.038** |
| CHF | 1.62 | 1.10 – 2.36 | **0.013** |
| Renal Failure | 1.14 | 0.81 – 1.61 | 0.448 |
| Stroke | 1.24 | 0.91 – 1.70 | 0.180 |
| Pulmonary Disease | 1.10 | 0.91 – 1.33 | 0.325 |
| PVD or HTN | 0.90 | 0.78 – 1.03 | 0.122 |
| Liver or PUD | 1.42 | 1.02 – 1.98 | **0.038** |
| Heme/Endo | 1.41 | 1.21 – 1.66 | **<0.001** |
| Diabetes | 0.94 | 0.80 – 1.11 | 0.446 |
| Cardiac | 0.96 | 0.80 – 1.17 | 0.704 |
| Psychiatric Disorder | 1.16 | 0.91 – 1.48 | 0.226 |
| Years Since Primary (per year increase; Ref 0) | 1.01 | 0.99 – 1.03 | 0.390 |
| Brain or Visceral Mets: Visceral vs None | 1.42 | 1.11 – 1.80 | **0.004** |
| Brain or Visceral Mets: Brain vs None | 0.79 | 0.48 – 1.29 | 0.343 |
| Brain or Visceral Mets: Brain and Visceral vs None | 1.49 | 0.83 – 2.67 | 0.183 |
| Primary: GI vs Breast | 2.74 | 2.13 – 3.52 | **<0.001** |
| Primary: Gyne vs Breast | 1.39 | 0.98 – 1.97 | 0.062 |
| Primary: HPB vs Breast | 2.46 | 1.78 – 3.40 | **<0.001** |
| Primary: Lung vs Breast | 2.72 | 2.22 – 3.33 | **<0.001** |
| Primary: Lymphoma vs Breast | 0.64 | 0.47 – 0.88 | **0.005** |
| Primary: Melanoma vs Breast | 2.80 | 2.04 – 3.85 | **<0.001** |
| Primary: Myeloma vs Breast | 0.64 | 0.51 – 0.82 | **<0.001** |
| Primary: Other vs Breast | 1.28 | 1.01 – 1.62 | **0.044** |
| Primary: Prostate vs Breast | 1.10 | 0.86 – 1.42 | 0.448 |
| Primary: Thyroid vs Breast | 0.74 | 0.47 – 1.16 | 0.193 |
| Primary: Urological vs Breast | 1.69 | 1.34 – 2.14 | **<0.001** |
| Spinal Level: Thoracolumbar vs Cervical | 0.92 | 0.82 – 1.02 | 0.115 |
| Intent of Chemo: Curative vs No Prior Chemo | 0.70 | 0.58 – 0.86 | **<0.001** |
| Intent of Chemo: Palliative vs No Prior Chemo | 1.00 | 0.85 – 1.17 | 0.993 |
| Lines of Chemo (Per additional line; ref 0 lines) | 1.12 | 1.01 – 1.25 | **0.036** |
| Intent of Rads: Curative vs No prior rads | 1.17 | 0.99 – 1.37 | 0.059 |
| Intent of Rads: Palliative vs No prior rads | 1.42 | 1.21 – 1.66 | **<0.001** |
| Neoadjuvant Spinal Radiation | 1.55 | 1.36 – 1.76 | **<0.001** |
| Paralysis or SCI | 1.43 | 1.26 – 1.62 | **<0.001** |
| Prior ICU Admission | 1.17 | 1.02 – 1.35 | **0.027** |
| Number of ED Visits (Per additional visit; ref 0 visits) | 1.08 | 1.05 – 1.11 | **<0.001** |

Baseline Survival at 6-month: 66.13%; 1-year: 49.08%; 1.5 years: 40.63%
